# Sex differences in epigenetic age in Mediterranean high longevity regions

**DOI:** 10.3389/fragi.2022.1007098

**Published:** 2022-11-23

**Authors:** Hannah-Ruth Engelbrecht, Sarah M. Merrill, Nicole Gladish, Julie L. MacIsaac, David T. S. Lin, Simone Ecker, Christina A. Chrysohoou, Giovanni M. Pes, Michael S. Kobor, David H. Rehkopf

**Affiliations:** ^1^ Edwin S. H. Leong Healthy Aging Program, Faculty of Medicine, University of British Columbia, Vancouver, BC, Canada; ^2^ Department of Medical Genetics, Faculty of Medicine, University of British Columbia, Vancouver, BC, Canada; ^3^ British Columbia Children’s Hospital Research Institute, University of British Columbia, Vancouver, BC, Canada; ^4^ Centre for Molecular Medicine and Therapeutics, University of British Columbia, Vancouver, BC, Canada; ^5^ Department of Epidemiology and Population Health, School of Medicine, Stanford University, Palo Alto, CA, United States; ^6^ UCL Cancer Institute, University College London, London, United Kingdom; ^7^ First Cardiology Clinic, School of Medicine, University of Athens, Athens, Greece; ^8^ Department of Clinical and Experimental Medicine, University of Sassari, Sassari, Italy

**Keywords:** blue zone, centenarian, epigenetic clock, biological age, telomere, biomarker, DNA methylation

## Abstract

Sex differences in aging manifest in disparities in disease prevalence, physical health, and lifespan, where women tend to have greater longevity relative to men. However, in the Mediterranean Blue Zones of Sardinia (Italy) and Ikaria (Greece) are regions of centenarian abundance, male-female centenarian ratios are approximately one, diverging from the typical trend and making these useful regions in which to study sex differences of the oldest old. Additionally, these regions can be investigated as examples of healthy aging relative to other populations. DNA methylation (DNAm)-based predictors have been developed to assess various health biomarkers, including biological age, Pace of Aging, serum interleukin-6 (IL-6), and telomere length. Epigenetic clocks are biological age predictors whose deviation from chronological age has been indicative of relative health differences between individuals, making these useful tools for interrogating these differences in aging. We assessed sex differences between the Horvath, Hannum, GrimAge, PhenoAge, Skin and Blood, and Pace of Aging predictors from individuals in two Mediterranean Blue Zones and found that men displayed positive epigenetic age acceleration (EAA) compared to women according to all clocks, with significantly greater rates according to GrimAge (β = 3.55; *p* = 1.22 × 10^−12^), Horvath (β = 1.07; *p* = 0.00378) and the Pace of Aging (β = 0.0344; *p* = 1.77 × 10^−08^). Other DNAm-based biomarkers findings indicated that men had lower DNAm-predicted serum IL-6 scores (β = -0.00301, *p* = 2.84 × 10^−12^), while women displayed higher DNAm-predicted proportions of regulatory T cells than men from the Blue Zone (*p* = 0.0150, 95% Confidence Interval [0.00131, 0.0117], Cohen’s d = 0.517). All clocks showed better correlations with chronological age in women from the Blue Zones than men, but all clocks showed large mean absolute errors (MAE >30 years) in both sexes, except for PhenoAge (MAE <5 years). Thus, despite their equal survival to older ages in these Mediterranean Blue Zones, men in these regions remain biologically older by most measured DNAm-derived metrics than women, with the exception of the IL-6 score and proportion of regulatory T cells.

## Introduction

Men and women seem to age differently—women tend to have greater morbidity and frailty in older ages but also live longer in most populations (Oksuzyan et al., 2008; Thinggaard et al., 2016; Hägg and Jylhävä, 2021). The diseases affecting aging men and women are also different (Hägg and Jylhävä, 2021). Chronic, non-lethal conditions and reduced health span disproportionately affect women more than men. Conversely, men display greater rates of heart disease, the leading cause of death globally, and higher incidence of non-reproductive cancers than women (Bots et al., 2017; Carmel, 2019; Meyer et al., 2020; Garmany et al., 2021). Given the lifespan and health span discrepancies between the sexes, studies of the oldest old (>80 years) provide useful opportunities to examine sex-related differences in healthy aging and biomarkers of the biological aging process, as the extended lifespans in these populations have allowed the accumulation of changes associated with aging (Puca et al., 2018).

While living to the age of 100 in most parts of the world is relatively unlikely, there exist four naturally occurring, validated, geographic regions of exceptional longevity, known as Blue Zones, with a high prevalence of centenarians (Buettner, 2012; Poulain et al., 2013): Sardinia (Italy), Ikaria (Greece), Nicoya Peninsula (Costa Rica) and Okinawa (Japan). These regions are geographically isolated, either being islands or separated from other regions by natural features such as mountains, and there is typically a traditional, “unmodernized” agricultural lifestyle observed (Poulain et al., 2011; Poulain and Mackowicz, 2021). A second unique feature in the Mediterranean Blue Zones, Ikaria and Sardinia (Poulain et al., 2011; Poulain and Mackowicz, 2021), is that equal proportions of men and women survive to extreme old age, in stark contrast to the preponderance of oldest old women commonly observed in most global populations (Passarino et al., 2002; Austad, 2011). While many aspects of lifestyle have been investigated in these locations, including psychological outlook, physical activity, and sociodemographic characterizations (Fastame et al., 2018; Hitchcott et al., 2018; Pes et al., 2018; Pes et al., 2020), there remains limited information regarding biological age differences between these men and women who survive to become centenarians more equally than the average global population. Biological age is a prediction of an individual’s chronological age that represents their functioning and health at a point in time, and has been used as a measure of well-being for many decades using a wide variety of health metrics (Furukawa et al., 1975; Rockwood et al., 2005; Jylhävä et al., 2017; Ji et al., 2021).

Epigenetic predictors have been developed to predict either chronological age or other biomarkers based on the DNA methylation (DNAm) levels in a particular combination of sites in the genome. DNAm, the addition of a methyl group to primarily cytosine-phosphate-guanine (CpG) sites of DNA, is a necessary part of the genomic regulatory suite that can change in response to external exposures and has both temporally stochastic changes and predictable changes at certain sites with increasing age (Horvath, 2013; Seale et al., 2022). Deviations between predicted epigenetic age and chronological age have been associated with differences in health, which may be related to behavioral and environmental exposures over the life course. With this conceptualization, lower predicted epigenetic age is typically indicative of better health, while higher predicted epigenetic age is indicative of poorer health (Jones et al., 2015). This has been demonstrated in many aging related conditions. For example, cancers and cardiovascular disease have been associated with increased epigenetic age (Perna et al., 2016; Soler-Botija et al., 2019). Epigenetic age predictors that are based on these consistent changes in DNAm are commonly termed epigenetic clocks. Many clocks exist, each using slightly different additive models of CpG site DNAm states to reach a prediction of epigenetic age, with some newer clocks incorporating other biological variables, such as DNAm-based predictors of serum protein levels (Bergsma and Rogaeva, 2020). In addition to epigenetic clocks, there are DNAm-based epigenetic predictors which estimate other age-associated biomarkers such as telomere length and cytokine levels, which have also been linked to health outcomes (Pusceddu et al., 2018; Villar-Fincheira et al., 2021).

Epigenetic clocks have been developed to detect different aspects of biological age using strict chronological age (Horvath, Hannum, Skin and Blood) (Hannum et al., 2013; Horvath, 2013; Horvath et al., 2018), mortality (GrimAge) (Lu A. T. et al., 2019), and general characteristics of aging such as frailty and increased heart disease risk (termed “phenotypic age”) (PhenoAge) (Levine et al., 2018). Furthermore, each epigenetic clock can be used to calculate a second measure, epigenetic age acceleration (EAA), the “ticking rate” of the clock. EAA is calculated by finding the residuals from the regression of the predicted epigenetic age on chronological age and can also be used to interrogate the rate at which aging is occurring. The aforementioned epigenetic clocks have been shown to discriminate between physical health and disease status (Quach et al., 2017; Hillary et al., 2020), and cognitive decline (Marioni et al., 2015), which also display sex differences in the aging process, positioning clocks as useful tools to investigate the sex differences in aging (Ahrenfeldt et al., 2019; Levine et al., 2021). According to a recently developed deep-learning based clock, epigenetic age in men accelerates sooner than in women (>55 years), supporting the ability of these epigenetic clocks to discriminate sex and age (Galkin et al., 2021). However, current epigenetic clocks were trained in predominantly middle-aged cohorts, with mean ages of approximately ∼50 years, and as such, their performance and behavior have not been thoroughly examined at the extreme upper end of life.

Additional DNAm-based biomarkers that complement epigenetic clocks have been developed, including a measure using DNAm to predict the annual rate of physiological decline (Pace of Aging) as a “cousin” measure to the clocks, a measure of telomere length (DNAmTL) (Lu A. T. et al., 2019), serum interleukin-6 (IL-6) (Stevenson et al., 2021), smoking status and score, and cell type proportions. The Pace of Aging was trained by using a longitudinal cohort of identically-aged individuals and monitoring the changes in DNAm at particular sites over time between 19 and 45 years of age. Thus, Pace of Aging represents a rate of physiological decline per annum (Belsky et al., 2022). This measure has been associated with health outcomes including dementia and frailty (Verschoor et al., 2021; Sugden et al., 2022), important features of the aging process, which are known to display sex differences (Anstey et al., 2021; Sindi et al., 2021). However, as the most recent version of the Pace of Aging has only been produced using individuals with a maximum age of 45 years, investigations into how this measure performs in a cohort of centenarians is needed to further characterize previously established associations and their relevance in the geroscience field.

Other biomarkers that have also been well-investigated in studies of both longevity and sex differences are telomere length and serum IL-6. Telomeres, the protective hexamer repeats that cap chromosomes, shorten over the lifespan, but are generally longer in women at all ages (Vaiserman and Krasnienkov, 2021). Studies of individuals from the Blue Zone in the Nicoya Peninsula in Costa Rica have indicated that Blue Zone residents have longer telomers than individuals from other regions in Costa Rica (Rehkopf et al., 2013). The longer telomeres observed in women have been postulated as both a mechanism and biomarker of longevity (Gardner et al., 2014; Öngel et al., 2021). However, telomere length has not been able to resolve aspects of healthy aging other than longevity (Arai et al., 2015). IL-6, an inflammatory cytokine, is known to increase with age, and is part of the chronic, low-grade, systemic inflammation that occurs with aging, known as inflammaging (Ferrucci and Fabbri, 2018). Centenarians have been found to possess lower inflammation measured by scores that include IL-6 as a main factor, potentially as a result of a counteracting anti-inflammaging response, and such findings have been associated with their better health (Arai et al., 2015; Minciullo et al., 2016). Smoking behavior has been associated with the risk of cardiovascular disease development in a sex-dependent manner (Vasiljevic et al., 2021), which has implications for the health of individuals. Cell-type differences predicted with DNAm have also been shown to differentiate amongst the oldest old in the Costa Rican Blue Zone, where a lower proportion of CD8^+^ memory T cells and higher proportion of naïve T cells were observed in Blue Zone residents compared to non-Blue Zone residents, indicating a younger immune system profile (McEwen et al., 2017). Similar to epigenetic clocks, the majority of these DNAm biomarker predictors have been developed or investigated predominantly in middle aged to young elderly cohorts (∼70 years), necessitating a characterization among the oldest old as well.

Focusing on the lack of characterization of epigenetic predictors among elderly populations, in these analyses we seek to characterize the performance of several predictors in a cohort of extremely elderly individuals from the Mediterranean Blue Zones, who remain understudied given their rarity. Initially, epigenetic clocks will be evaluated to assess at a molecular level whether long-surviving men from the Blue Zones display a comparable biological age to long-surviving women, as expected by their documented similar mortality rates. Subsequently, we use DNAm-based predictors of telomere length (DNAmTL), smoking score (EpiSmokER), and serum IL-6 (DNAm IL-6 score) which may indicate health differences in the oldest old from regions of exceptional longevity, reporting any differences related to sex (Lu A. T. et al., 2019; Bollepalli et al., 2019; Stevenson et al., 2021).

## Methods

### Cohort recruitment, sample collection, and data collection

A subset of participants from two separate studies, one in the Ikaria Blue Zone in Greece and one in the Sardinia Blue Zone in Italy, were selected for DNAm analysis (Pes et al., 2020; Foscolou et al., 2021) (Table 1). Given that these regions are in relatively close proximity geographically when compared to the other Blue Zones in Costa Rica and Japan, have relatively similar climates, diets, and cultures, and the same male survival phenomenon is observed, we combined the Sardinian and Ikarian Blue Zone residents into one cohort to represent the Mediterranean Blue Zones (Poulain et al., 2021; Pes et al., 2022). Venous blood was collected from participants with informed consent from both Blue Zone regions as part of each study. Age at time of blood collection and self-reported sex were used as the variables of interest in this study. DNA was extracted from the blood samples and shipped to the University of British Columbia (Vancouver, British Columbia, Canada). DNA was bisfulfite treated using the EZ-DNA methylation kit (Zymo Research, CA, United States), and DNAm data were measured using the MethylationEPIC BeadChip array (“EPIC”, Illumina, San Diego, CA, United States) according to manufacturer’s protocols. This study was approved by the Committee on Human Subjects at Stanford University.

**TABLE 1:** Age, age range, and number of participants by region and sex.

Mediterranean blue zone	Sample size (% female)	Mean age in years (range)
Ikaria, Greece	45 (67)	89 (63–107)
Sardinia, Italy	49 (49)	89 (71–104)
Total	94 (57)	89 (63–107)

While matching Italian and Greek control participants from beyond the Blue Zone regions were recruited using the same process, the participants recruited from the Blue Zone regions were markedly older than the participants recruited from matching control regions, and there were far fewer control region participants than Blue Zone residents (Supplementary Figure S1). Given that health and DNAm can change quite dramatically in the extreme end of life, that age acts as a risk factor for most health-related outcomes (Niccoli and Partridge, 2012), and given that the sample size was underpowered for any statistical analysis, control participants could not be included in any statistical comparisons.

### Data normalization, preprocessing

The Illumina BeadArray Data (IDAT) files from the array run were imported into R 4.0.3 (R Core Team, 2022). Poorly performing samples which were identified as outliers based on the ratio of the log2 unmethylated to methylated intensity of the control probes were removed (*n* = 3). Functional normalization using the *preprocessFunnorm* function from *minfi* was performed to adjust background and probe type intensity and (Fortin et al., 2014). Any samples identified by *detectOutlier* from the *lumi* package (Du et al., 2008), *pcout* from *wateRmelon* (Pidsley et al., 2013), *locfdr* from the *locFDR* package (Hannum et al., 2013), or *pfilter* from wateRmelon (Pidsley et al., 2013), were deemed outliers and removed (*n* = 4). DNAm-based sex prediction was performed using the *getSex* function from the *minfi* suite based on DNAm data on the X and Y chromosomes (Fortin et al., 2014). In addition to this measure, self-reported sex and DNAm-based log2 intensity of XY chromosome probes estimated sex were confirmed against each other, which led to the removal of 7 sex-mismatched samples. A second confirmation of sex was completed by using a k-means clustering approach of the DNAm from the 16,839 probes on the X and Y chromosomes, and no further mismatches were identified. The normalized cohort consisted of 112 individuals from the Blue Zone and control regions. Further processing involved the removal of a selection of probes from the EPIC array as is standard in the field, including SNP probes (*n* = 13,110), known polymorphic probes (*n* = 23,906), sex chromosome probes (*n* = 17,344), cross-hybridizing probes (*n* = 9,441) (Price et al., 2013), probes represented by three beads or less, failed detection in ≥1% of samples, or had a detection *p*-value > 1 × 10^−16^ (*n* = 59,154) (Pidsley et al., 2016; Fortin et al., 2017). The *ComBat* function from the *sva* package (Leek et al., 2019, 2012) was used for technical correction of plate, row position, and chip.

### Epigenetic age prediction and DNAm-derived biological proxy measure calculation

After all processing steps were completed, the resulting DNAm beta matrix was uploaded to the DNA Methylation Age Calculator^1^ (Horvath, 2013) for epigenetic age prediction. When uploaded to the DNA Methylation Age Calculator, “Normalize Data” and “Advanced Analysis” were selected as options. When selecting the “Advanced Analysis” option, the DNA Methylation Age Calculator provides the Horvath, Hannum, Skin and Blood, PhenoAge, GrimAge, and DNAmTL predictions. This same DNAm data was used to calculate the Pace of Aging using the *DunedinPACE* predictor R package with the *PoAmProjector* function^2^ (Belsky et al., 2022). Epigenetic age acceleration (EAA) was calculated for the epigenetic clocks (Horvath, Hannum, Skin and Blood, PhenoAge and GrimAge) by obtaining the residuals from the linear model: Epigenetic age ∼ Chronological age. The features of the predictors used is provided in Supplementary Table S1.

In addition to the explicitly age-related DNAm-based biomarkers obtained above, two other DNAm-based biomarkers were calculated which predicts smoking and serum IL-6 levels. Smoking status and score provide an indication of smoking behavior and status and were calculated using EpiSmokEr in R (Bollepalli et al., 2019): Smoking status was calculated based on the DNAm of 121 CpGs identified by Bollepati et al., and smoking score was calculated based on DNAm of 187 previously identified CpGs (Elliott et al., 2014). The DNAm-based serum IL-6 score was calculated in R using a weighted score from the DNAm status of 12 CpGs (Stevenson et al., 2021). A summary of the epigenetic clocks, biomarkers, and their main features is provided in Supplementary Table S1. The data preprocessing and normalization steps selected for the prediction of epigenetic age were assessed based on the prediction concordance between chronological age-prediction clocks (Supplemental Figures 2 and 3).

### Prediction and transformation of cell type proportions

DNAm is a key component of cell identity, and the source of DNAm data in this study is a heterogenous tissue, venous whole blood. To resolve and account for cell-based DNAm signal in downstream analyses, cell type deconvolution (a means of predicting cell type proportions in the tissue sample) was completed with constraint projections using the IDOL package in R for extended blood cell types (Koestler et al., 2016; Gervin et al., 2019; Salas et al., 2022). Prior to use in linear models, the cell type proportions were transformed using robust isometric principal components (PCs) (Filzmoser et al., 2009).

### Assessment of epigenetic clock performance

Pearson’s correlation coefficients (*r*) between the chronological age of individuals and their predicted epigenetic age across the Horvath, Hannum, GrimAge, PhenoAge, and Skin and Blood epigenetic clocks were compared for men and women of the Mediterranean Blue Zones. As the remaining epigenetic predictors (Pace of Aging, DNAmTL, cell type proportions, IL-6 and smoking score) do not measure age in years, they were not included in these comparisons of year-based outcomes. The mean absolute error and maximum absolute error (MAE and MaxAE) were calculated for each epigenetic clock’s age prediction and chronological age using the *mae* and *maxae* functions in the R package *mlr3measures* (Lang et al., 2019).

### Statistical model of biomarker comparisons

For the purposes of this study, the statistical significance threshold was defined as a *p*-value of 0.05 Bonferroni-corrected for nine predictor comparisons (*p* = 5.56 × 10^–03^). Linear regression was performed to compare differences between the sexes. In order to account for the low sample size and ensure the models were not overfitted, the covariates included: sex, age, and the first four PCs accounting for 80% of cell type, with female sex being considered the reference: Biomarker ∼ Sex + Age + Cell type PC1 + Cell type PC2 + Cell type PC3 + Cell type PC4.

### Cell type proportion comparisons

The 12 cell type proportions were compared between men and women using t-tests of mean proportion per cell type. Tukey’s Honest Significant Difference test and resulting adjusted *p*-value were used to assess the significance of cell types with a *p*-value slightly <0.05.

## Results

### Epigenetic clocks perform differently between the sexes amongst the oldest old in the Blue Zones

**FIGURE 1:**
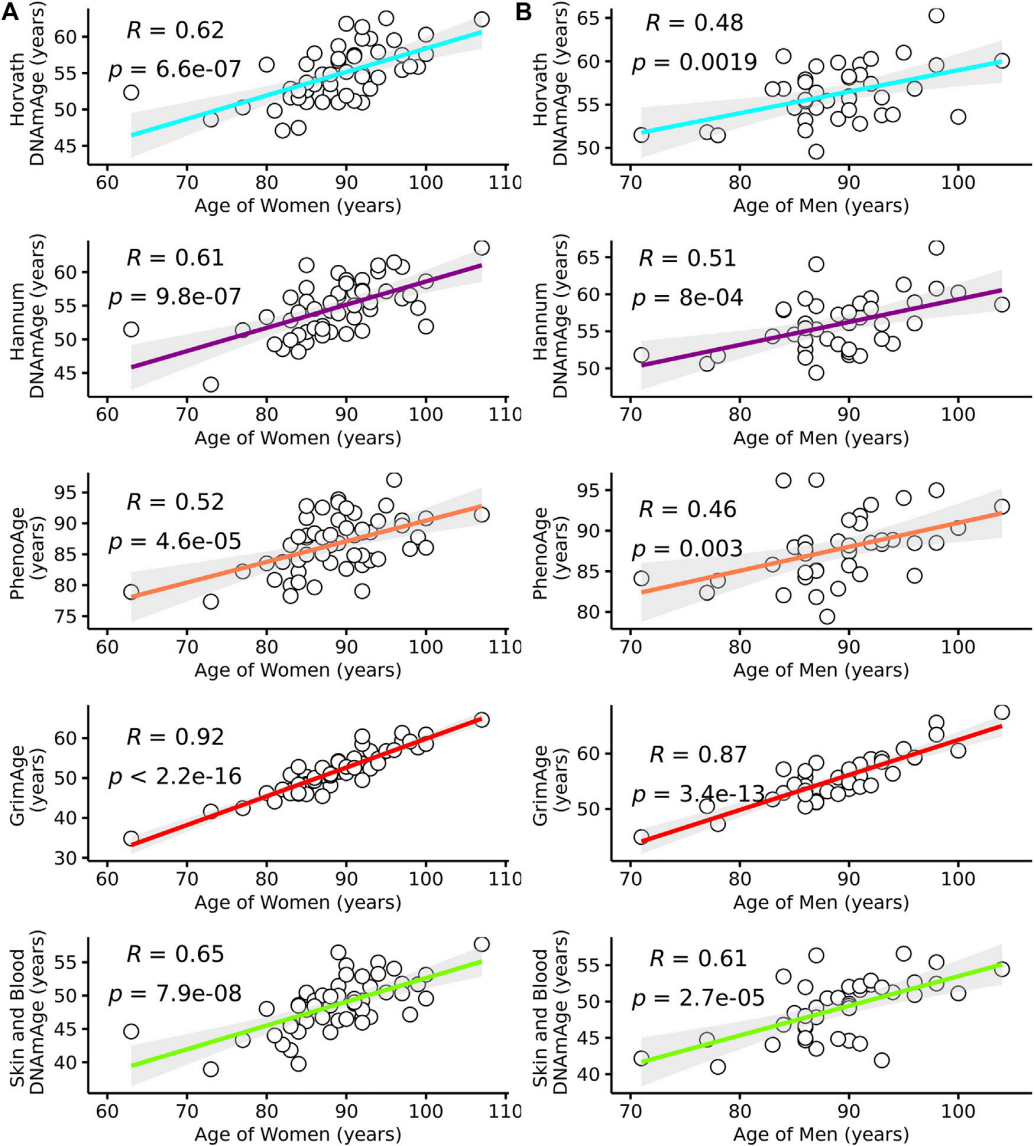
Pearson’s correlation coefficients (*r*) between predicted epigenetic age and chronological age for several epigenetic clocks in **(A)** Female and **(B)** Male residents of the Mediterranean Blue Zones.

**FIGURE 2:**
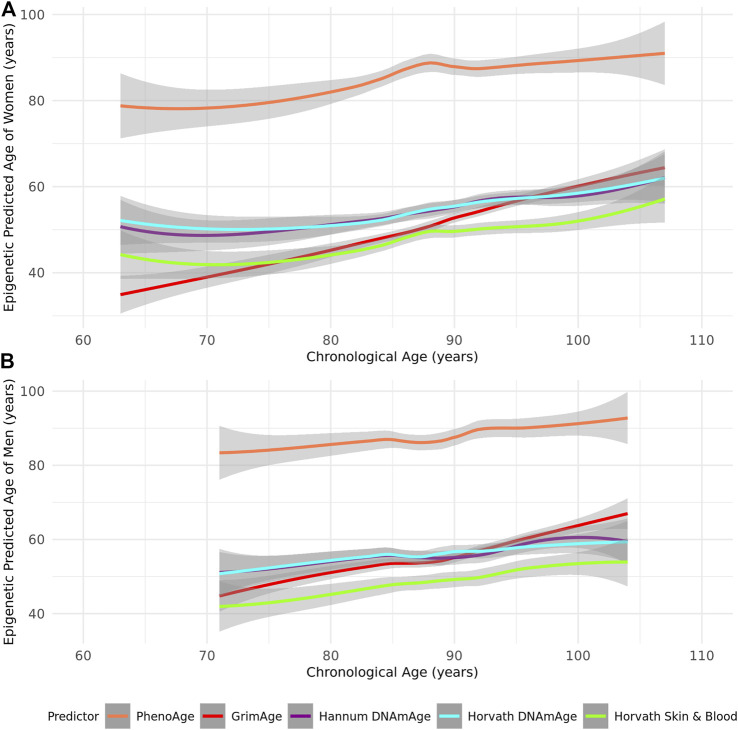
A Loess-smoothed curve of epigenetic predicted age with 95% confidence interval in grey against chronological age in years according to the PhenoAge, GrimAge, Hannum, Horvath, and Skin and Blood epigenetic age predictors in **(A)** female and **(B)** male residents of the Mediterranean Blue Zones.

As most epigenetic clocks are trained on approximately middle-aged individuals (Supplementary Table S1), we first assessed the performance of the Horvath, Hannum, Skin and Blood, GrimAge, and PhenoAge epigenetic clocks in this group of the oldest old. We identified that all epigenetic age predictors had higher correlations with chronological age in women (*r* = 0.52–0.92 in women and *r* = 0.46–0.87 in men across the tested clocks) (Table 2; Figure 1). PhenoAge demonstrated the lowest correlation with chronological age (*r* < 0.5 in men and women), while GrimAge outperformed the other clocks with strong correlations among both sexes (*r* > 0.8) (Table 2; Figure 1). However, the mean and maximum errors were consistently quite large for both sexes across all clocks (>30 years of error) except PhenoAge, which showed mean and maximum errors <5 years for both men and women, the smallest differences between predicted and chronological age (Table 2; Figure 2).

**TABLE 2:** Pearson’s correlations (*r*), Mean Absolute Error, and Maximum Absolute Error of the Horvath, Hannum, PhenoAge, GrimAge, and Skin and Blood epigenetic clocks in Men and Women from the Mediterranean Blue Zones.

Epigenetic clock	Women (*n* = 54)			Men (*n* = 40)		
	R	Mean absolute error (years)	Maximum absolute error (years)	R	Mean absolute error (years)	Maximum absolute error (years)
Horvath	0.617	33.9	44.6	0.476	32.9	46.4
Hannum	0.610	33.9	48.1	0.510	33.2	45.4
PhenoAge	0.525	4.97	15.9	0.457	4.47	13.1
GrimAge	0.923	36.97	42.5	0.869	33.5	39.5
Skin and Blood	0.655	40.0	50.9	0.612	40.1	51.1

### Epigenetic age acceleration evident in oldest old men compared to oldest old women from the Blue Zones

**FIGURE 3:**
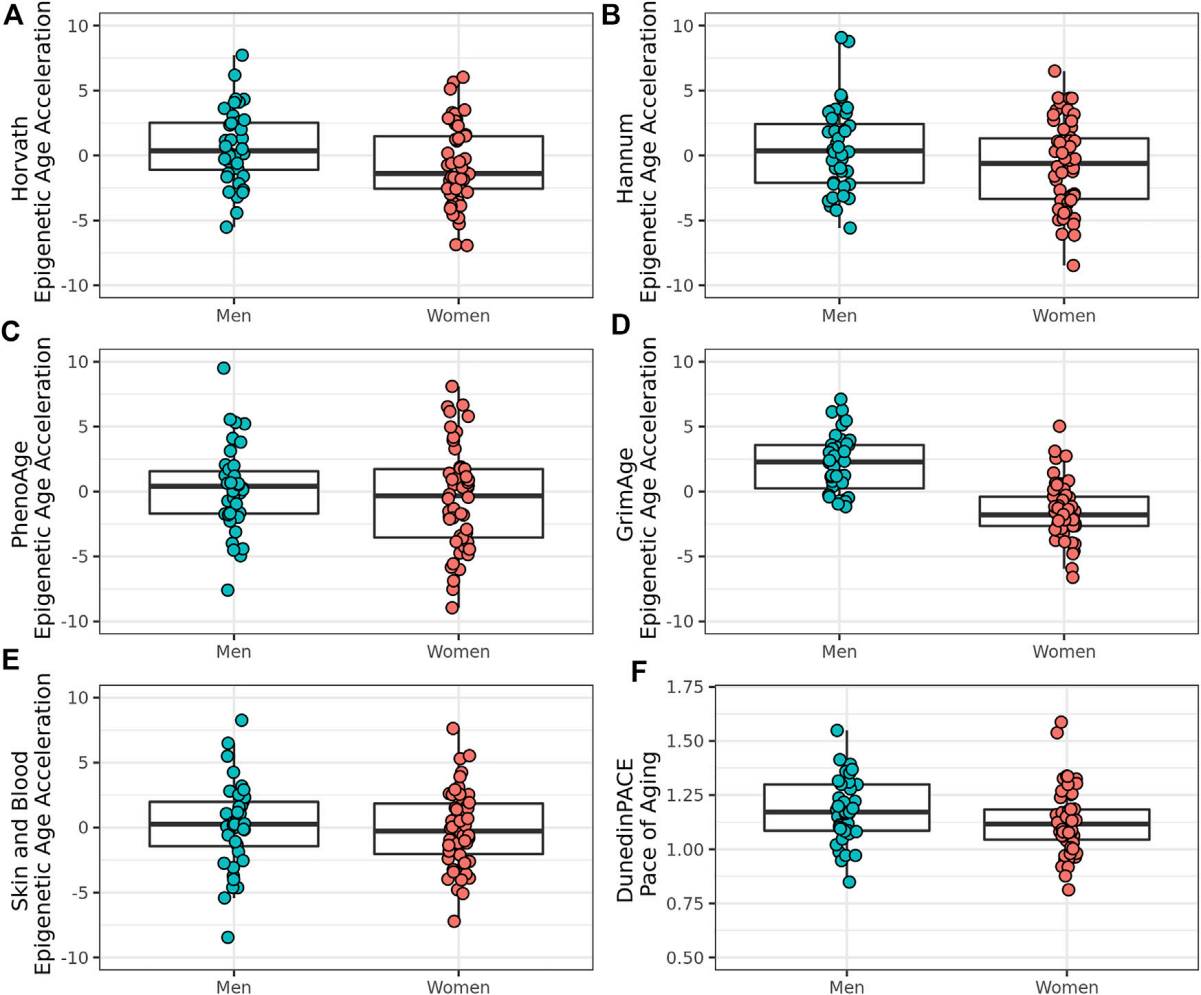
Epigenetic age acceleration and Pace of Aging in male and female residents of the Mediterranean Blue Zones, according to the **(A)** Horvath, **(B)** Hannum, **(C)** PhenoAge, **(D)** GrimAge, **(E)** Skin and Blood, and **(F)** Pace of Aging predictors.

To explore sex differences in biological age measures in this group of oldest old individuals from the Mediterranean Blue Zones, we examined the differences in the Pace of Aging measure and EAA across the Horvath, Hannum, GrimAge, PhenoAge, and Skin and Blood clocks. Men showed greater EAA than women across all epigenetic age predictors, with significant differences observed for the GrimAge (Adjusted *R* ^*2*^ = 0.501, *F* (6, 87) = 16.6, *p* = 1.22 × 10^–12^, Cohen’s *f* ^*2*^ = 1.00), Horvath (Adjusted *R* ^*2*^ = 0.139, F (6,87) = 3.50, *p* = 0.00378, Cohen’s *f* ^*2*^ = 0.161), and Pace of Aging predictors (Adjusted *R* ^*2*^ = 0.383, *F* (6, 87) = 9.23, *p* = 1.77 × 10^–08^, Cohen’s *f* ^*2*^ = 0.620) (Figure 3; Table 3).

**TABLE 3:** Positive Epigenetic Age Acceleration (EAA) is evident in men from the Blue Zone relative to women from the Blue Zone. The coefficient estimate indicates the linear model β coefficient associated with the specified independent variable in brackets, and Cohen’s *f* ^2^ effect size based on the Adjusted *R* ^*2*^ of the linear model. Bolded *p*-values indicate significance (corrected *p*-value < 0.00556).

	Coefficient estimate β (men)	Multiple *R* ^*2*^	Adjusted *R* ^*2*^	Cohen’s *f* ^*2*^	*p*-value
Horvath EAA	1.07	0.195	0.139	0.161	**0.00378**
Hannum EAA	1.42	0.171	0.114	0.129	0.0103
PhenoAge EAA	1.30	0.177	0.120	0.137	0.00810
GrimAge EAA	3.55	0.533	0.501	1.00	**1.22 × 10** ^**−12**^
Skin and Blood EAA	0.249	0.0147	−0.0531	−0.0504	0.970
Pace of Aging	0.0344	0.429	0.383	0.620	**1.77 × 10** ^**−08**^
DNAmTL	−0.0443	0.346	0.301	0.430	**1.28 × 10** ^**−06**^
Smoking Score	1.00	0.103	0.0410	0.0428	0.140
Serum IL-6	−0.00301	0.524	0.491	0.964	**2.84 × 10** ^**−12**^

### Regulatory T cell proportions higher in women from the Mediterranean Blue Zones

**FIGURE 4:**
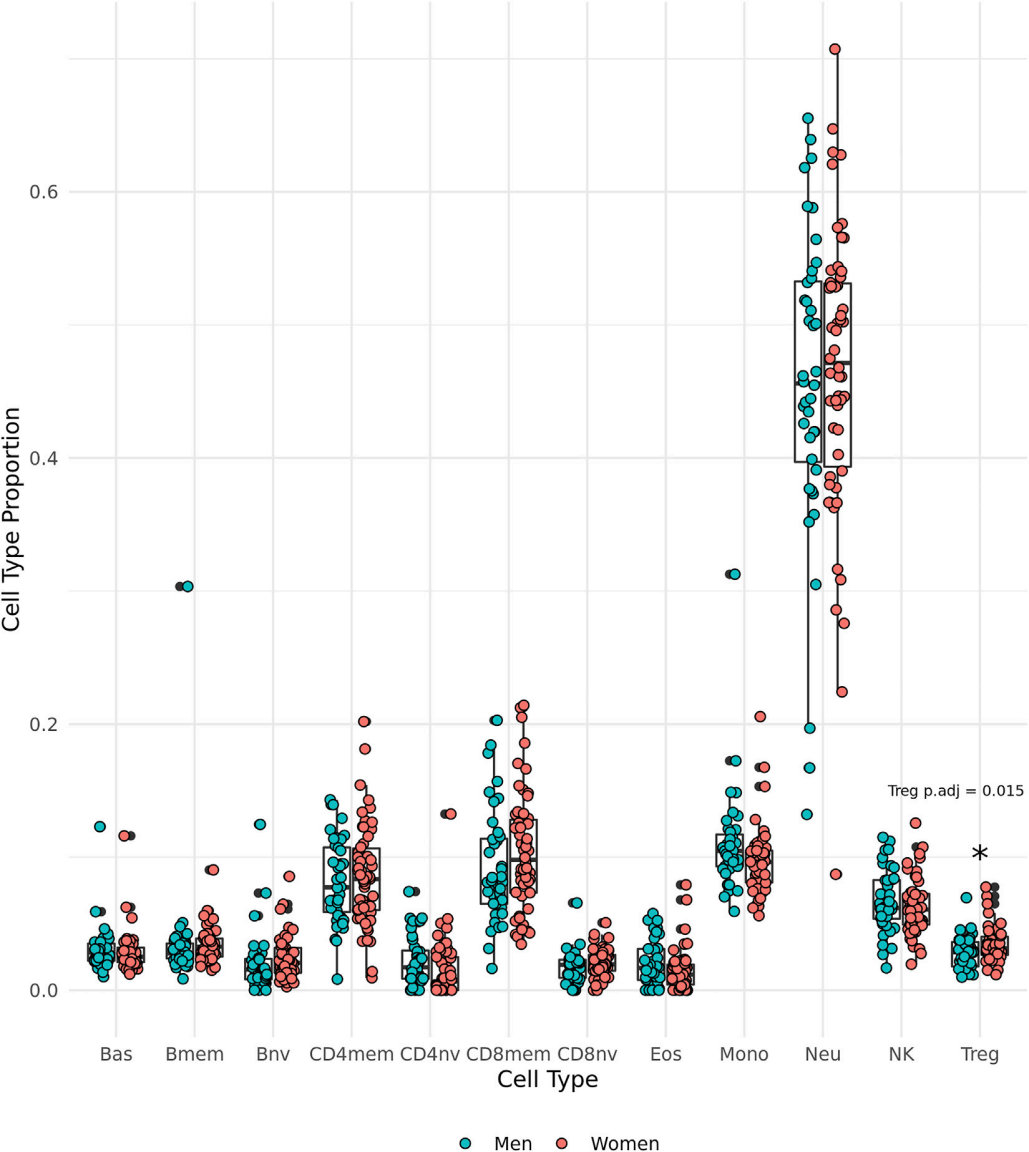
Epigenetically predicted cell type proportion by sex amongst the oldest old in the Mediterranean Blue Zones, including basophils (Bas), memory B cells (Bmem), naïve B cells (Bnv), memory CD4 T cells (CD4mem), memory CD8 T cells (CD8mem), naïve CD8 T cells (CD8nv), eosinophils (Eos), Monocytes (Mono), Neutrophils (Neu), Natural Killer cells (NK), and regulatory T cells (Treg).

DNAm-predicted cell type proportions were used to make comparisons between the sexes. We observed that the oldest old women have significantly greater proportions of regulatory T cells (Tregs) than the oldest old men in the group (*t* (85.85) = 2.49, *p* = 0.0150, 95% CI [0.00131, 0.0117], Cohen’s *d* = 0.517) (Figure 4).

### Smoking score, predicted IL-6 score, and telomere length differ between the sexes in Mediterranean Blue Zones

**FIGURE 5:**
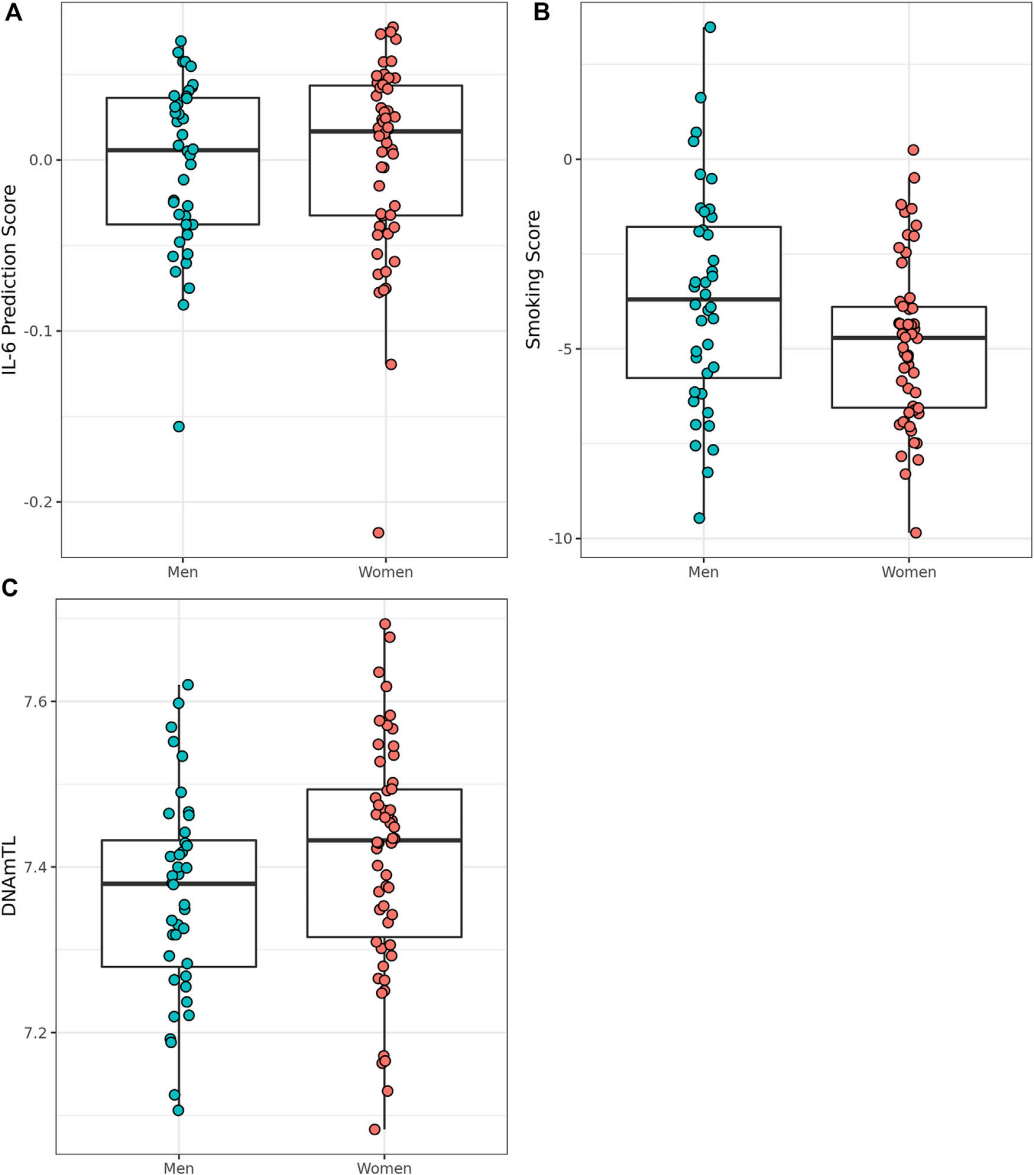
Epigenetically predicted biomarkers by sex amongst the oldest old in the Mediterranean Blue Zones, **(A)** serum IL-6, **(B)** Smoking Score, and **(C)** leukocyte telomere length (DNAmTL).

We assessed smoking status and smoking score in the Mediterranean Blue Zone residents to determine whether the scores differed between the sexes of the oldest. Of the 94 Mediterranean Blue Zone residents, four were predicted to have never smoked, while 90 participants were classified as “former smokers” using DNAm-based detection methods in EpiSmokER. Men were not more likely to have smoked than women according to comparisons of smoking score. To examine potential signals of inflammaging, we assessed sex differences in predicted serum IL-6 scores between oldest old men and women from the Blue Zones. Men were predicted to have a lower serum IL-6 score than women (Adjusted *R* ^2^ = 0.49, *F* (6, 87) = 15.94, *p* = 2.84 × 10^–12^, Cohen’s *f* ^*2*^ = 0.96) in this group. The oldest old men also displayed shorter telomere length than women according to DNAmTL predictions (Adjusted *R* ^*2*^ *=* 0.30, *F* (6, 87) = 7.66, *p* = 1.28 × 10^–06^, Cohen’s *f* ^*2*^ = 0.04) (Figure 5).

## Discussion

The aims of our study were to characterize the performance of the epigenetic clocks within a group of the oldest old, and secondly, to investigate markers of biological age in a unique longevous population, located in the Mediterranean Blue Zones, to develop a more comprehensive understanding of the sex differences that occur with extreme age. Men in these longevity regions, Sardinia and Ikaria, tend to survive into extreme old age at approximately the same rate as women, which suggest that they might have a health advantage relative to men from beyond these regions (Poulain et al., 2021). We assessed the performance of the epigenetic clocks in the Mediterranean Blue Zones, and all epigenetic clocks were found to have better correlations with chronological age in women compared to men, though a high error rate was found across all clocks except PhenoAge for both men and women. We then examined sex differences of EAA and observed that men had increased EAA, Pace of Aging, and smoking score relative to women from the Mediterranean Blue Zones (Horvath, 2013; Lu A. T. et al., 2019; Belsky et al., 2022). Though we observed minimal differences in cell type proportions, there was the exception in the proportion of Tregs where women were predicted to have higher levels than men. Additionally, we observed differences in DNAm-based biomarkers of telomere length and serum IL-6 where men were predicted to have shorter telomeres and lower levels of IL-6 relative to women. In general, DNAm-derived predictors of age and age-related biomarkers indicate men in these Mediterranean Blue Zones age biologically faster than women, despite their apparent survival advantage relative to men from other regions.

An examination of the performance of the epigenetic clocks indicated an overall poor performance of the epigenetic clocks in the Mediterranean Blue Zone group of the oldest old. The clocks had either poor correlations of epigenetic and chronological age, and/or large MAE and MaxAE rates according to every epigenetic age predictor assessed in both sexes. GrimAge, despite having a large error of approximately 30 years, along with the Horvath, Hannum, and Skin and Blood epigenetic clocks, also had the highest correlation with chronological age in men and women from the Mediterranean Blue Zones, which is comparable with epigenetic clock performance in other elderly cohorts (Ecker and Beck, 2019). We observed that all correlations of epigenetic and chronological age were stronger in women than men from the Mediterranean Blue Zones. Of all the tested clocks, GrimAge had the strongest correlation in women, indicating the aging health trajectory in this group of oldest old women is likely well-accounted for by this clock. Interestingly, all correlations between epigenetic age and chronological age were consistently lower in men from this region. The clocks tested here were not developed using populations with evidence of male longevity, and while each clock captures a unique aspect of the broader aging process, they currently do so in a manner that reflects female longevity. It may be that potential DNAm differences associated with the male longevity phenomenon of the Mediterranean Blue Zones are not captured by the current epigenetic clocks, which may indicate why increased male EAA, as is typical in most populations, is observed (Horvath et al., 2016).

The best performing clock by correlation was GrimAge, which is the only predictor tested here that incorporates a DNAm-based prediction of smoking pack-years (Lu A. T. et al., 2019). As observed *via* the EpiSmokER results, the vast majority of this cohort have smoked at some point in their lives, though this is very expected in this population, as smoking is quite common in the areas of Italy and Greece where Sardinia and Ikaria are located (Lugo et al., 2013; Christopoulou et al., 2021; Gallus et al., 2021). In fact, increased GrimAge, Horvath, and the Pace of Aging, which showed greater EAA and pace of aging in men, have all been associated with smoking (Klopack et al., 2022). GrimAge may display the highest correlation with chronological age because it is accounting for the molecular signature of well-established modifier of DNAm and health traits that may have sex-specific effects (Koo et al., 2021), even without sex-differential rates of smoking (Maas et al., 2020), and was developed using the oldest training cohort (Lu A. T. et al., 2019). It has also been observed that GrimAge EAA has more associations with other features of older biological age than PhenoAge, Horvath, and Hannum EAA, such as increased polypharmacy, lower grip strength, and slower walking speed, many of which display sex-differences themselves (Hägg and Jylhävä, 2021; McCrory et al., 2021). This suggests that GrimAge may be better suited to resolving differences in health status in the oldest old, rather than accurately predicting chronological age of the oldest old.

In contrast to the GrimAge EAA findings, we observed low correlations for PhenoAge and chronological age, yet also the lowest margins of error. PhenoAge clock training also included serum protein and cell counts in its training, but it has little similarity to those markers of aging used by GrimAge. Additionally, PhenoAge does not use DNAm-based predictions of these serum proteins, as it was trained in a two-stage process, which defined phenotypic age (based on serum proteins and white blood cell counts) in one cohort, and then trained to detect that phenotypic age using CpGs in a second cohort. The serum proteins used by these epigenetic clocks (GrimAge and PhenoAge) are distinct, with minimal similarity, barring C-reactive protein, which is used by both (Levine et al., 2018; Lu A. T. et al., 2019). The low error rates of PhenoAge in this cohort of oldest old individuals suggest that PhenoAge may be better suited to predicting oldest old age, and may be detecting general physiological trends that occur in the aging process. However, given the low correlation between predicted epigenetic and chronological age, PhenoAge is less able to resolve differences in health amongst the oldest old.

While women are, on average, less physically well than age-matched male counterparts, very few measures explain the lifespan gap that occurs. Epigenetic age has been well-associated with sex-specific features of aging, including frailty (Breitling et al., 2016; Maddock et al., 2019), cardiovascular disease (Roetker et al., 2018), and cognitive decline (Maddock et al., 2019; Vaccarino et al., 2021). Both telomere length and epigenetic age, however, better reflect the survival of women, although these molecular measures are not well correlated and cannot easily be compared to each other (Marioni et al., 2016). Women generally have longer telomeres than men at all ages (Gardner et al., 2014; Tucker, 2019), and men are found to have greater epigenetic age and EAA than women, with this effect becoming more pronounced at advanced ages (Horvath et al., 2016; Galkin et al., 2021; Kankaanpää et al., 2021). In the current Mediterranean Blue Zone population with equal male-female survival to advanced age, the commonly observed female aging advantage was also observed here by slower EAA in the most discriminating epigenetic clock, as well as longer predicted DNAm-derived telomere length. Additionally, the Pace of Aging predictor—one of the most current and well-validated predictors (Belsky et al., 2022, 2020)—produced higher scores among men relative to women, indicating that these molecular biomarkers of biological age may reflect the generally observed female survival to older ages. However, this is surprising given the hypothesized similarity of men’s and women’s biological aging, based on their similar survival rates in the Mediterranean Blue Zones. Therefore, it is reasonable to conjecture that these DNAm-derived measures do not capture the factors driving the protective effect evidenced by male survival to oldest old ages in the Mediterranean Blue Zones (Crimmins et al., 2021; Kankaanpää et al., 2021).

Alternatively, we did observe a lower DNAm-predicted serum IL-6 score in men than in women. Unlike EAA or DNAm-derived telomere length, this finding aligns with the predicted extended survival effect of men in these unique areas, as lower levels of serum IL-6 have been associated with healthy aging based on cognitive and physical ability (Puzianowska-Kuźnicka et al., 2016), thus potentially reflecting extended male survival. IL-6 is a cytokine secreted by several cell types during inflammation, which acts as a signaling molecule (Papanicolaou, 2000; Frasca and Blomberg, 2016; Kaur et al., 2020), and importantly, increases with age (Ershler, 1993). Previous research regarding the immune system of Sardinian centenarians has suggested a younger immune profile based on serum neopterin concentrations (a compound produced by macrophages in response to infection), which indicates lesser inflammation than expected for their age (Sotgia et al., 2017). The lower predicted IL-6 observed in this study in men from the Mediterranean Blue Zones further contributes to what may be an indicator of the survival advantage in men of this Blue Zone cohort, and suggests a modified inflammaging phenotype, specifically, rather than a general biological aging advantage.

Previous research interrogating immune system differences in the Nicoya Peninsula Blue Zone observed lower proportions of CD8^+^ memory T cells and higher proportions of naive T cells, suggesting a general younger immune profile (McEwen et al., 2017). In this study, men from the Mediterranean Blue Zones were predicted to have lower proportions of regulatory T cells (Tregs) than women. Tregs are usually involved in maintaining a level of homeostasis within the immune system by suppressing the immune response and promoting self-tolerance, but at older ages these cells tend to accumulate. Tregs play important protective roles in limiting autoimmune disorders (Tamosiuniene et al., 2018; Goodman et al., 2020), but functional Tregs are susceptible to both epigenetic alteration of the usually-expressed *FOXP3* gene (Shu et al., 2017) and impaired estrogen signaling (Goodman et al., 2020, 2014), which may impair immunity and increase susceptibility to autoimmune diseases. The accumulation of dysregulated functional Tregs may contribute to immune system dysregulation by overly suppressing immune responses (Raynor et al., 2012; Klein and Flanagan, 2016; Müller et al., 2019; Churov et al., 2020; Rocamora-Reverte et al., 2021). Thus, the lower proportion of these Tregs in men from the Mediterranean Blue Zones compared to women may provide biological support for the similar male-female survival rate into old age and suggests an avenue worth investigating into male survival.

Sex differences in DNAm are known to exist (Grant et al., 2022), but the epigenetic clocks are trained to use age, aging phenotype, and mortality-related CpG sites as input, with the assumption that the age-related sites are likely the same in both sexes across the age spectrum. As found by McCrory et al. (2020), however, cardiovascular and metabolic conditions were more strongly associated with higher PhenoAge EAA in women, while Horvath EAA had greater associations with metabolic conditions in men. It is possible that in these understudied oldest old groups that age and age-related disease affected CpG sites become more sex-specific, or represent different aspects of the aging process that are sex-specific (McCrory et al., 2020). It is uncommon to have a very large cohort where oldest old men and women from a relatively homogenous environment can be compared, and ostensibly further studies of such cohorts may indicate that health phenotype differences between the sexes are sufficiently great that specific tools may be required to interrogate the complexity of different aspects of aging, either health trajectory or general biological age prediction. This is highlighted by the PhenoAge findings discussed above—that age-related CpG sites may remain similar, hence the low error metrics, but health-distinguishing sites may differ, and thus the low correlations appear.

While there are several limitations common to many studies of the oldest old, notably the small sample size, the lack of an age-matched control group, and a lack of health status data for this group of individuals that would further elucidate the findings of this study, we present suggestive data that add to the growing body of evidence that aging of the oldest old men and women cannot be analyzed adequately with extrapolations from tools designed in younger adults. Given the small number of control participants and their much younger age, it was not possible to make comparisons regarding possible sex differences within control regions and the Mediterranean Blue Zones. We were unable to measure any counteracting anti-inflammaging response in these long-lived individuals, and given that we only have DNA-predicted Treg proportion information, we cannot determine whether the difference is functionally relevant. Finally, the relatively poor performance of the clocks observed here may indicate that, as the epigenetic clocks assume a linear relationship between chronological age and epigenetic age, it may be that at the oldest old age range, the predicted relationship may not be linear making these clocks inaccurate in this age group (Snir et al., 2019). Despite the above limitations, this study has the strength of equal numbers of oldest old men and women from unique areas, the Mediterranean Blue Zones. In these regions where healthy aging, manifested as longevity, is prevalent, it is possible to investigate health span in order to garner insights into the aging process at the upper end of life.

This study analyzed the performance of increasingly popular biological aging measures, epigenetic clocks, and then compared these and other DNAm-derived health predictors in a rare population of equally long-surviving men and women from the Mediterranean Blue Zones—regions of centenarian abundance. We observed oldest old men had accelerated biological aging and shorter telomere length in comparison to oldest old women in these Blue Zones according to what we detected as the most precise predictors in this group, despite the equal longevity of the sexes in these regions. We also observed lower predicted IL-6 score in men and higher Treg proportion in women from the Mediterranean Blue Zone, which were both potential indicators of the survival advantage in men in these regions through specific immunological pathways perhaps not fully captured by DNAm-derived telomere length and epigenetic clocks. However, the relatively poor performance of all epigenetic clocks does indicate that these tools, currently trained using approximately middle-aged cohorts, performed inadequately, and epigenetic clocks with a focus on the oldest old should be developed to better assess health of the elderly and to capture sex-specific drivers of EAA. We found compelling evidence the epigenetic clocks support the female longevity phenomenon, even in regions of equal male longevity, but we also found evidence these predictors may fail to capture different aspects of the aging process amongst the oldest old. To this end, further research is required to understand the sex-dependent lifespan and health span gaps, which could be elucidated by an inflammation and immune-focused direction to interrogate the survival benefits of men in the Mediterranean Blue Zones.

## Data Availability

The datasets generated for this study are available on request to the corresponding authors.

## Footnotes

1 DNA Methylation Age Calculator: https://dnamage.genetics.ucla.edu/new

2 DunedinPACE package: https://github.com/danbelsky/DunedinPACE

## References

[B1] Ahrenfeldt L. J. Möller S. Thinggaard M. Christensen K. Lindahl-Jacobsen R. (2019). Sex differences in comorbidity and frailty in Europe. Int. J. Public Health 64, 1025–1036. 10.1007/s00038-019-01270-9 31236603 PMC7237816

[B2] Anstey K. J. Peters R. Mortby M. E. Kiely K. M. Eramudugolla R. Cherbuin N. (2021). Association of sex differences in dementia risk factors with sex differences in memory decline in a population-based cohort spanning 20–76 years. Sci. Rep. 11, 7710–10. 10.1038/s41598-021-86397-7 33833259 PMC8032756

[B3] Arai Y. Martin-Ruiz C. M. Takayama M. Abe Y. Takebayashi T. Koyasu S. (2015). Inflammation, but not telomere length, predicts successful ageing at extreme old age: A longitudinal study of semi-supercentenarians. EBioMedicine 2, 1549–1558. 10.1016/j.ebiom.2015.07.029 26629551 PMC4634197

[B4] Austad S. N. (2011). “Sex differences in longevity and aging,” in Handbook of the biology of aging. Handbooks of aging. Editors Masoro E. J. Austad S. N.. Seventh Edition (San Diego: Academic Press), 479–495. 10.1016/B978-0-12-378638-8.00023-3

[B5] Belsky D. W. Caspi A. Arseneault L. Baccarelli A. Corcoran D. L. Gao X. (2020). Quantification of the pace of biological aging in humans through a blood test, the DunedinPoAm DNA methylation algorithm. eLife 9, e54870. 10.7554/eLife.54870 32367804 PMC7282814

[B6] Belsky D. W. Caspi A. Corcoran D. L. Sugden K. Poulton R. Arseneault L. (2022). Quantification of the pace of biological aging in humans through a blood test, the DunedinPoAm DNA methylation algorithm. eLife 11, e54870. 10.7554/eLife.54870 PMC7282814 32367804

[B7] Bergsma T. Rogaeva E. (2020). DNA methylation clocks and their predictive capacity for aging phenotypes and healthspan. Neurosci. Insights 15, 2633105520942221. 10.1177/2633105520942221 32743556 PMC7376380

[B8] Bollepalli S. Korhonen T. Kaprio J. Anders S. Ollikainen M. (2019). EpiSmokEr: A robust classifier to determine smoking status from DNA methylation data. Epigenomics 11, 1469–1486. 10.2217/epi-2019-0206 31466478

[B9] Bots S. H. Peters S. A. E. Woodward M. (2017). Sex differences in coronary heart disease and stroke mortality: A global assessment of the effect of ageing between 1980 and 2010. BMJ Glob. Health 2, e000298. 10.1136/bmjgh-2017-000298 PMC5435266 28589033

[B10] Breitling L. P. Saum K.-U. Perna L. Schöttker B. Holleczek B. Brenner H. (2016). Frailty is associated with the epigenetic clock but not with telomere length in a German cohort. Clin. Epigenetics 8, 21. 10.1186/s13148-016-0186-5 26925173 PMC4768341

[B11] Buettner D. (2012). 9 lessons for living longer from the people who’ve lived the longest. Second Edition. Washington, DC: National Geographic.The Blue Zones

[B12] Carmel S. (2019). Health and well-being in late life: Gender differences worldwide. Front. Med. 6, 218. 10.3389/fmed.2019.00218 PMC6795677 31649931

[B13] Christopoulou R. Mavropoulos G. Voucharas G. (2021). The Greek smoking epidemic from a life-course perspective. J. Public Health, fdab342. fdab342. 10.1093/pubmed/fdab342 PMC9715303 34498081

[B14] Churov A. V. Mamashov K. Y. Novitskaia A. V. (2020). Homeostasis and the functional roles of CD4+ Treg cells in aging. Immunol. Lett. 226, 83–89. 10.1016/j.imlet.2020.07.004 32717201

[B15] Crimmins E. M. Thyagarajan B. Levine M. E. Weir D. R. Faul J. (2021). Associations of age sex race/ethnicity and education with 13 epigenetic clocks in a nationally representative U.S. sample: The Health and Retirement Study. J. Gerontol. A Biol. Sci. Med. Sci. 76, 1117–1123. 10.1093/GERONA/GLAB016 33453106 PMC8140049

[B16] Du P. Kibbe W. A. Lin S. M. (2008). lumi: a pipeline for processing Illumina microarray. Bioinformatics 24, 1547–1548. 10.1093/bioinformatics/btn224 18467348

[B17] Ecker S. Beck S. (2019). The epigenetic clock: A molecular crystal ball for human aging? Aging 11, 833–835. 10.18632/aging.101712 30669120 PMC6366965

[B18] Elliott H. R. Tillin T. McArdle W. L. Ho K. Duggirala A. Frayling T. M. (2014). Differences in smoking associated DNA methylation patterns in South Asians and Europeans. Clin. Epigenetics 6, 4. 10.1186/1868-7083-6-4 24485148 PMC3915234

[B19] Ershler W. B. (1993). Interleukin-6: A cytokine for gerontologists. J. Am. Geriatr. Soc. 41, 176–181. 10.1111/j.1532-5415.1993.tb02054.x 8426042

[B20] Fastame M. Hitchcott P. Mulas I. Ruiu M. Penna M. (2018). Resilience in elders of the Sardinian Blue Zone: An explorative study. Behav. Sci. 8, 30. 10.3390/bs8030030 29495425 PMC5867483

[B21] Ferrucci L. Fabbri E. (2018). Inflammageing: Chronic inflammation in ageing, cardiovascular disease, and frailty. Nat. Rev. Cardiol. 15, 505–522. 10.1038/s41569-018-0064-2 30065258 PMC6146930

[B22] Filzmoser P. Hron K. Reimann C. (2009). Principal component analysis for compositional data with outliers. Environmetrics 20, 621–632. 10.1002/env.966

[B23] Fortin J.-P. Labbe A. Lemire M. Zanke B. W. Hudson T. J. Fertig E. J. (2014). Functional normalization of 450k methylation array data improves replication in large cancer studies. Genome Biol. 15, 503. 10.1186/s13059-014-0503-2 25599564 PMC4283580

[B24] Fortin J. P. Triche T. J. Hansen K. D. (2017). Preprocessing, normalization and integration of the Illumina HumanMethylationEPIC array with minfi. Bioinformatics 33, 558–560. 10.1093/BIOINFORMATICS/BTW691 28035024 PMC5408810

[B25] Foscolou A. Chrysohoou C. Dimitriadis K. Masoura K. Vogiatzi G. Gkotzamanis V. (2021). The association of healthy aging with multimorbidity: IKARIA study. Nutrients 13, 1386. 10.3390/nu13041386 33924100 PMC8074281

[B26] Frasca D. Blomberg B. B. (2016). Inflammaging decreases adaptive and innate immune responses in mice and humans. Biogerontology 17, 7–19. 10.1007/s10522-015-9578-8 25921609 PMC4626429

[B27] Furukawa T. Inoue M. Kajiya F. Inada H. Takasugi S. Fukui S. (1975). Assessment of biological age by multiple regression analysis. J. Gerontol. 30, 422–434. 10.1093/geronj/30.4.422 1141673

[B28] Galkin F. Mamoshina P. Kochetov K. Sidorenko D. Zhavoronkov A. (2021). DeepMAge: A methylation aging clock developed with deep learning. Aging Dis. 12, 1252–1262. 10.14336/AD.2020.1202 34341706 PMC8279523

[B29] Gallus S. Lugo A. Liu X. Behrakis P. Boffi R. Bosetti C. (2021). Who smokes in europe? Data from 12 European countries in the TackSHS survey 2017–2018). J. Epidemiol. 31, 145–151. 10.2188/jea.JE20190344 32249267 PMC7813769

[B30] Gardner M. Bann D. Wiley L. Cooper R. Hardy R. Nitsch D. (2014). Gender and telomere length: Systematic review and meta-analysis. Exp. Gerontol. 51, 15–27. 10.1016/j.exger.2013.12.004 24365661 PMC4523138

[B31] Garmany A. Yamada S. Terzic A. (2021). Longevity leap: Mind the healthspan gap. NPJ Regen. Med. 6, 57. 10.1038/s41536-021-00169-5 34556664 PMC8460831

[B32] Gervin K. Salas L. A. Bakulski K. M. Zelm M. C. Koestler D. C. Wiencke J. K. (2019). Systematic evaluation and validation of reference and library selection methods for deconvolution of cord blood DNA methylation data. Clin. Epigenetics 11, 125. 10.1186/s13148-019-0717-y 31455416 PMC6712867

[B33] Goodman W. A. Bedoyan S. M. Havran H. L. Richardson B. Cameron M. J. Pizarro T. T. (2020). Impaired estrogen signaling underlies regulatory T cell loss-of-function in the chronically inflamed intestine. Proc. Natl. Acad. Sci. U. S. A. 117, 17166–17176. 10.1073/pnas.2002266117 32632016 PMC7382259

[B34] Goodman W. A. Garg R. R. Reuter B. K. Mattioli B. Rissman E. F. Pizarro T. T. (2014). Loss of estrogen-mediated immunoprotection underlies female gender bias in experimental Crohn’s-like ileitis. Mucosal Immunol. 7, 1255–1265. 10.1038/mi.2014.15 24621993 PMC4139459

[B35] Grant O. A. Wang Y. Kumari M. Zabet N. R. Schalkwyk L. (2022). Characterising sex differences of autosomal DNA methylation in whole blood using the Illumina EPIC array. Clin. Epigenetics 141 14, 62–16. 10.1186/S13148-022-01279-7 PMC9107695 35568878

[B36] Hägg S. Jylhävä J. (2021). Sex differences in biological aging with a focus on human studies. eLife 10, e63425–e63427. 10.7554/eLife.63425 33982659 PMC8118651

[B37] Hannum G. Guinney J. Zhao L. Zhang L. Hughes G. Sadda S. (2013). Genome-wide methylation profiles reveal quantitative views of human aging rates. Mol. Cell 49, 359–367. 10.1016/j.molcel.2012.10.016 23177740 PMC3780611

[B38] Hillary R. F. Stevenson A. J. McCartney D. L. Campbell A. Walker R. M. Howard D. M. (2020). Epigenetic measures of ageing predict the prevalence and incidence of leading causes of death and disease burden. Clin. Epigenetics 12, 115. 10.1186/S13148-020-00905-6 32736664 PMC7394682

[B39] Hitchcott P. K. Fastame M. C. Penna M. P. (2018). More to Blue Zones than long life: Positive psychological characteristics. Health Risk Soc. 20, 163–181. 10.1080/13698575.2018.1496233

[B40] Horvath S. (2013). DNA methylation age of human tissues and cell types. Genome Biol. 14, 115. 10.1186/gb-2013-14-10-r115 24138928 PMC4015143

[B41] Horvath S. Gurven M. Levine M. E. Trumble B. C. Kaplan H. Allayee H. (2016). An epigenetic clock analysis of race/ethnicity, sex, and coronary heart disease. Genome Biol. 17, 171. 10.1186/s13059-016-1030-0 27511193 PMC4980791

[B42] Horvath S. Oshima J. Martin G. M. Lu A. T. Quach A. Cohen H. (2018). Epigenetic clock for skin and blood cells applied to Hutchinson Gilford Progeria Syndrome and *ex vivo* studies. Aging 10, 1758–1775. 10.18632/aging.101508 30048243 PMC6075434

[B43] Ji L. Jazwinski S. M. Kim S. (2021). Frailty and biological age. Ann. Geriatr. Med. Res. 25, 141–149. 10.4235/agmr.21.0080 34399574 PMC8497950

[B44] Jones M. J. Goodman S. J. Kobor M. S. (2015). DNA methylation and healthy human aging. Aging Cell 14, 924–932. 10.1111/acel.12349 25913071 PMC4693469

[B45] Jylhävä J. Pedersen N. L. Hägg S. (2017). Biological age predictors. eBioMedicine 21, 29–36. 10.1016/J.EBIOM.2017.03.046 28396265 PMC5514388

[B46] Kankaanpää A. Tolvanen A. Saikkonen P. Heikkinen A. Laakkonen E. K. Kaprio J. (2021). Do epigenetic clocks provide explanations for sex differences in life span? A cross-sectional twin study. J. Gerontol. A Biol. Sci. Med. Sci. 77, 1898–1906. 10.1093/gerona/glab337 PMC9434475 34752604

[B47] Kaur S. Bansal Y. Kumar R. Bansal G. (2020). A panoramic review of IL-6: Structure, pathophysiological roles and inhibitors. Bioorg. Med. Chem. 28, 115327. 10.1016/j.bmc.2020.115327 31992476

[B48] Klein S. L. Flanagan K. L. (2016). Sex differences in immune responses. Nat. Rev. Immunol. 16, 626–638. 10.1038/nri.2016.90 27546235

[B49] Klopack E. T. Carroll J. E. Cole S. W. Seeman T. E. Crimmins E. M. (2022). Lifetime exposure to smoking, epigenetic aging, and morbidity and mortality in older adults. Clin. Epigenetics 14, 72. 10.1186/s13148-022-01286-8 35643537 PMC9148451

[B50] Koestler D. C. Jones M. J. Usset J. Christensen B. C. Butler R. A. Kobor M. S. (2016). Improving cell mixture deconvolution by identifying optimal DNA methylation libraries (IDOL). BMC Bioinforma. 17, 120. 10.1186/s12859-016-0943-7 PMC4782368 26956433

[B51] Koo H.-K. Morrow J. Kachroo P. Tantisira K. Weiss S. T. Hersh C. P. (2021). Sex-specific associations with DNA methylation in lung tissue demonstrate smoking interactions. Epigenetics 16, 692–703. 10.1080/15592294.2020.1819662 32962511 PMC8143227

[B52] Lang M. Binder M. Richter J. Schratz P. Pfisterer F. Coors S. (2019). mlr3: A modern object-oriented machine learning framework in R. J. Open Source Softw. 4, 1903. 10.21105/joss.01903

[B53] Leek J. T. Johnson W. E. Parker H. S. Fertig E. J. Jaffe A. E. Storey J. D. (2019). sva: surrogate variable analysis.

[B54] Leek J. T. Johnson W. E. Parker H. S. Jaffe A. E. Storey J. D. (2012). The sva package for removing batch effects and other unwanted variation in high-throughput experiments. Bioinformatics 28 (6), 882–883. 10.1093/bioinformatics/bts034 22257669 PMC3307112

[B55] Levine D. A. Gross A. L. Briceño E. M. Tilton N. Giordani B. J. Sussman J. B. (2021). Sex differences in cognitive decline among US adults. JAMA Netw. Open 4, 210169. 10.1001/JAMANETWORKOPEN.2021.0169 PMC7907956 33630089

[B56] Levine M. E. Lu A. T. Quach A. Chen B. H. Assimes T. L. Bandinelli S. (2018). An epigenetic biomarker of aging for lifespan and healthspan. Aging 10, 573–591. 10.18632/aging.101414 29676998 PMC5940111

[B57] Lu A. T. Quach A. Wilson J. G. Reiner A. P. Aviv A. Raj K. (2019a). DNA methylation GrimAge strongly predicts lifespan and healthspan. Aging 11, 303–327. 10.18632/aging.101684 30669119 PMC6366976

[B58] Lu A. T. Seeboth A. Tsai P. C. Sun D. Quach A. Reiner A.P. (2019b). DNA methylation-based estimator of telomere length. Aging (Albany. NY) 11, 5895–5923. 10.18632/AGING.102173 31422385 PMC6738410

[B59] Lugo A. Vecchia C. Boccia S. Murisic B. Gallus S. (2013). Patterns of smoking prevalence among the elderly in Europe. Int. J. Environ. Res. Public Health 10, 4418–4431. 10.3390/ijerph10094418 24048208 PMC3799502

[B60] Maas S. C. E. Mens M. M. J. Kühnel B. van Meurs J. B. J. Uitterlinden A. G. Peters A. (2020). Smoking-related changes in DNA methylation and gene expression are associated with cardio-metabolic traits. Clin. Epigenetics 12, 157. 10.1186/s13148-020-00951-0 33092652 PMC7579899

[B61] Maddock J. Castillo-Fernandez J. Wong A. Cooper R. Richards M. Ong K. K. (2019). DNA methylation age and physical and cognitive ageing. J. Gerontol. A Biol. Sci. Med. Sci. glz246. 10.1093/gerona/glz246 PMC8414926 31630156

[B62] Marioni R. E. Harris S. E. Shah S. McRae A. F. Zglinicki T. Martin-Ruiz C. (2016). The epigenetic clock and telomere length are independently associated with chronological age and mortality. Int. J. Epidemiol. 45, 424–432. 10.1093/IJE/DYW041 27075770 PMC4864882

[B63] Marioni R. E. Shah S. McRae A. F. Ritchie S. J. Muniz-Terrera G. Harris S. E. (2015). The epigenetic clock is correlated with physical and cognitive fitness in the Lothian Birth Cohort 1936. Int. J. Epidemiol. 44, 1388–1396. 10.1093/ije/dyu277 25617346 PMC4588858

[B64] McCrory C. Fiorito G. Hernandez B. Polidoro S. O’Halloran A. M. Hever A. (2021). GrimAge outperforms other epigenetic clocks in the prediction of age-related clinical phenotypes and all-cause mortality. J. Gerontol. A Biol. Sci. Med. Sci. 76, 741–749. 10.1093/gerona/glaa286 33211845 PMC8087266

[B65] McCrory C. Fiorito G. McLoughlin S. Polidoro S. Cheallaigh C. N. Bourke N. (2020). Epigenetic clocks and allostatic load reveal potential sex-specific drivers of biological aging. J. Gerontol. A Biol. Sci. Med. Sci. 75, 495–503. 10.1093/gerona/glz241 31603985

[B66] McEwen L. M. Morin A. M. Edgar R. D. MacIsaac J. L. Jones M. J. Dow W. H. (2017). Differential DNA methylation and lymphocyte proportions in a Costa Rican high longevity region. Epigenetics Chromatin 10, 21. 10.1186/s13072-017-0128-2 28465725 PMC5408416

[B67] Meyer A. C. Drefahl S. Ahlbom A. Lambe M. Modig K. (2020). Trends in life expectancy: Did the gap between the healthy and the ill widen or close? BMC Med. 18, 41. 10.1186/s12916-020-01514-z 32192480 PMC7082956

[B68] Minciullo P. L. Catalano A. Mandraffino G. Casciaro M. Crucitti A. Maltese G. (2016). Inflammaging and anti-inflammaging: The role of cytokines in extreme longevity. Arch. Immunol. Ther. Exp. 64, 111–126. 10.1007/s00005-015-0377-3 26658771

[B69] Müller L. Di Benedetto S. Pawelec G. (2019). “The immune system and its dysregulation with aging,” in Biochemistry and cell biology of ageing: Part II clinical Science Subcellular biochemistry. Editors Harris J. R. Korolchuk V. I. (Singapore: Springer), 21–43. 10.1007/978-981-13-3681-2_2 30888648

[B70] Niccoli T. Partridge L. (2012). Ageing as a risk factor for disease. Curr. Biol. 22, 741–752. 10.1016/J.CUB.2012.07.024 22975005

[B71] Oksuzyan A. Juel K. Vaupel J. W. Christensen K. (2008). Men: Good health and high mortality. Sex differences in health and aging. Aging Clin. Exp. Res. 20, 91–102. 10.1007/BF03324754 18431075 PMC3629373

[B72] Öngel M. E. Yıldız C. Akpınaroğlu C. Yilmaz B. Özilgen M. (2021). Why women may live longer than men do? A telomere-length regulated and diet-based entropic assessment. Clin. Nutr. 40, 1186–1191. 10.1016/j.clnu.2020.07.030 32807581

[B73] Papanicolaou D. A. Vgontzas A. N. (2000). Interleukin-6: The endocrine cytokine. J. Clin. Endocrinol. Metab. 85, 1331–1333. 10.1210/jcem.85.3.6582 10720086

[B74] Passarino G. Calignano C. Vallone A. Franceschi C. Jeune B. Robine J. (2002). Male/female ratio in centenarians: A possible role played by population genetic structure. Exp. Gerontol. 37, 1283–1289. 10.1016/S0531-5565(02)00140-7 12470842

[B75] Perna L. Zhang Y. Mons U. Holleczek B. Saum K.-U. Brenner H. (2016). Epigenetic age acceleration predicts cancer, cardiovascular, and all-cause mortality in a German case cohort. Clin. Epigenetics 8, 64. 10.1186/s13148-016-0228-z 27274774 PMC4891876

[B76] Pes G. M. Dore M. P. Errigo A. Poulain M. (2018). Analysis of physical activity among free–living nonagenarians from a Sardinian longevous population. J. Aging Phys. Act. 26, 254–258. 10.1123/japa.2017-0088 28714795

[B77] Pes G. M. Dore M. P. Tsofliou F. Poulain M. (2022). Diet and longevity in the Blue Zones: A set-and-forget issue? Maturitas 164, 31–37. 10.1016/j.maturitas.2022.06.004 35780634

[B78] Pes G. M. Errigo A. Tedde P. Dore M. P. (2020). Sociodemographic, clinical and functional profile of nonagenarians from two areas of Sardinia characterized by distinct longevity levels. Rejuvenation Res. 23, 341–348. 10.1089/rej.2018.2129 31613707

[B79] Pidsley R. Wong C. Y. Volta M. Lunnon K. Mill J. Schalkwyk L. C. (2013). A data-driven approach to preprocessing Illumina 450K methylation array data. BMC Genomics 14, 293. 10.1186/1471-2164-14-293 23631413 PMC3769145

[B80] Pidsley R. Zotenko E. Peters T. J. Lawrence M. G. Risbridger G. P. Molloy P. (2016). Critical evaluation of the Illumina MethylationEPIC BeadChip microarray for whole-genome DNA methylation profiling. Genome Biol. 17, 208. 10.1186/s13059-016-1066-1 27717381 PMC5055731

[B84] Poulain M. Pes G. Salaris L. (2011). A population where men live as long as women: Villagrande Strisaili, Sardinia. J. Aging Res. 2011, 153756. 10.4061/2011/153756 22132327 PMC3205712

[B82] Poulain M. Herm A. Pes G. (2013). The Blue Zones: Areas of exceptional longevity around the world. Vienna Yearb. Popul. Res. Vol. 11, 87–108. 10.1553/populationyearbook2013s87

[B81] Poulain M. Herm A. Errigo A. Chrysohoou C. Legrand R. Passarino G. (2021). Specific features of the oldest old from the longevity Blue Zones in Ikaria and Sardinia. Mech. Ageing Dev. 198, 111543. 10.1016/J.MAD.2021.111543 34265327

[B83] Poulain M. Mackowicz J. (Editors) (2021). Positive ageing and learning from centenarians: Living longer and better (London: Routledge). 10.4324/9781003162216

[B85] Price E. M. Cotton A. M. Lam L. L. Farré P. Emberly E. Brown C. J. (2013). Additional annotation enhances potential for biologically-relevant analysis of the Illumina Infinium HumanMethylation450 BeadChip array. Epigenetics Chromatin 6, 4. 10.1186/1756-8935-6-4 23452981 PMC3740789

[B86] Puca A. A. Spinelli C. Accardi G. Villa F. Caruso C. (2018). Centenarians as a model to discover genetic and epigenetic signatures of healthy ageing. Mech. Ageing Dev. 174, 95–102. 10.1016/j.mad.2017.10.004 29096878

[B87] Pusceddu I. Kleber M. Delgado G. Herrmann W. März W. Herrmann M. (2018). Telomere length and mortality in the ludwigshafen risk and cardiovascular health study. PLoS One 13, e0198373. 10.1371/JOURNAL.PONE.0198373 29920523 PMC6007915

[B88] Puzianowska-Kuźnicka M. Owczarz M. Wieczorowska-Tobis K. Nadrowski P. Chudek J. Slusarczyk P. (2016). Interleukin-6 and C-reactive protein, successful aging, and mortality: The PolSenior study. Immun. Ageing 13, 21. 10.1186/s12979-016-0076-x 27274758 PMC4891873

[B89] Quach A. Levine M. E. Tanaka T. Lu A. T. Chen B. H. Ferrucci L. (2017). Epigenetic clock analysis of diet, exercise, education, and lifestyle factors. Aging 9, 419–446. 10.18632/aging.101168 28198702 PMC5361673

[B90] R Core Team (2022). R Foundation for Statistical Computing. Vienna, Austria.

[B91] Raynor J. Lages C. S. Shehata H. Hildeman D. A. Chougnet C. A. (2012). Homeostasis and function of regulatory T cells in aging. Curr. Opin. Immunol. 24, 482–487. 10.1016/j.coi.2012.04.005 22560294 PMC3419320

[B92] Rehkopf D. H. Dow W. H. Rosero-Bixby L. Lin J. Epel E. S. Blackburn E. H. (2013). Longer leukocyte telomere length in Costa Rica’s Nicoya Peninsula: A population-based study. Exp. Gerontol. 48, 1266–1273. 10.1016/J.EXGER.2013.08.005 23988653 PMC3819141

[B93] Rocamora-Reverte L. Melzer F. L. Würzner R. Weinberger B. (2021). The complex role of regulatory T cells in immunity and aging. Front. Immunol. 11, 616949. 10.3389/fimmu.2020.616949 33584708 PMC7873351

[B94] Rockwood K. Song X. MacKnight C. Bergman H. Hogan D. B. McDowell I. (2005). A global clinical measure of fitness and frailty in elderly people. C. Can. Med. Assoc. J. 173, 489–495. 10.1503/CMAJ.050051 PMC1188185 16129869

[B95] Roetker N. S. Pankow J. S. Bressler J. Morrison A. C. Boerwinkle E. (2018). Prospective study of epigenetic age acceleration and incidence of cardiovascular disease outcomes in the ARIC Study Atherosclerosis Risk in Communities). Circ. Genom. Precis. Med. 11, e001937. 10.1161/CIRCGEN.117.001937 29555670 PMC5863591

[B96] Salas L. A. Zhang Z. Koestler D. C. Butler R. A. Hansen H. M. Molinaro A. M. (2022). Enhanced cell deconvolution of peripheral blood using DNA methylation for high-resolution immune profiling. Nat. Commun. 13, 761. 10.1038/s41467-021-27864-7 35140201 PMC8828780

[B97] Seale K. Horvath S. Teschendorff A. Eynon N. Voisin S. (2022). Making sense of the ageing methylome. Nat. Rev. Genet. 23, 585–605. 10.1038/s41576-022-00477-6 35501397

[B98] Shu Y. Hu Q. Long H. Chang C. Lu Q. Xiao R. (2017). Epigenetic variability of CD4+CD25+ Tregs Contributes to the pathogenesis of autoimmune diseases. Clin. Rev. Allergy Immunol. 52, 260–272. 10.1007/s12016-016-8590-3 27687891

[B99] Sindi S. Kåreholt I. Ngandu T. Rosenberg A. Kulmala J. Johansson L. (2021). Sex differences in dementia and response to a lifestyle intervention: Evidence from Nordic population-based studies and a prevention trial. Alzheimer’s Dement. 17, 1166–1178. 10.1002/ALZ.12279 34255432 PMC8361986

[B100] Snir S. Farrell C. Pellegrini M. (2019). Human epigenetic ageing is logarithmic with time across the entire lifespan. Epigenetics 14, 912–926. 10.1080/15592294.2019.1623634 31138013 PMC6691990

[B101] Soler-Botija C. Gálvez-Montón C. Bayés-Genís A. (2019). Epigenetic biomarkers in cardiovascular diseases. Front. Genet. 10, 950. 10.3389/fgene.2019.00950 31649728 PMC6795132

[B102] Sotgia S. Zinellu A. Mangoni A. A. Serra R. Pintus G. Caruso C. (2017). Cellular immune activation in Sardinian middle-aged, older adults and centenarians. Exp. Gerontol. 99, 133–137. 10.1016/j.exger.2017.10.005 29024722

[B103] Stevenson A. J. Gadd D. A. Hillary R. F. McCartney D. L. Campbell A. Walker R. M. (2021). Creating and validating a DNA methylation-based proxy for Interleukin-6. J. Gerontol. A Biol. Sci. Med. Sci. 76, 2284–2292. 10.1093/gerona/glab046 33595649 PMC8599002

[B104] Sugden K. Caspi A. Elliott M. L. Bourassa K. J. Chamarti K. Corcoran D. L. (2022). Association of pace of aging measured by blood-based DNA methylation with age-related cognitive impairment and dementia. Neurology 99, e1402–e1413. 10.1212/WNL.000000000020089810.1212/WNL.0000000000200898 35794023 PMC9576288

[B105] Tamosiuniene R. Manouvakhova O. Mesange P. Saito T. Qian J. Sanyal M. (2018). Dominant role for regulatory T cells in protecting females against pulmonary hypertension. Circ. Res. 122, 1689–1702. 10.1161/CIRCRESAHA.117.312058 29545367 PMC5993601

[B106] Thinggaard M. McGue M. Jeune B. Osler M. Vaupel J. W. Christensen K. (2016). Survival prognosis in very old adults. J. Am. Geriatr. Soc. 64, 81–88. 10.1111/JGS.13838 26782855 PMC4749674

[B107] Tucker L. A. (2019). Serum and dietary folate and vitamin B12 levels account for differences in cellular aging: Evidence based on telomere findings in 5581 U.S. Adults. Oxid. Med. Cell. Longev. 2019, 4358717. 10.1155/2019/4358717 31687079 PMC6800923

[B108] Vaccarino V. Huang M. Wang Z. Hui Q. Shah A. J. Goldberg J. (2021). Epigenetic age acceleration and cognitive decline: A twin study. J. Gerontol. A Biol. Sci. Med. Sci. 76, 1854–1863. 10.1093/gerona/glab047 33606025 PMC8436988

[B109] Vaiserman A. Krasnienkov D. (2021). Telomere length as a marker of biological age: State-of-the-art open issues and future perspectives. Front. Genet. 11, 630186. 10.3389/fgene.2020.630186 33552142 PMC7859450

[B110] Vasiljevic Z. Scarpone M. Bergami M. Yoon J. van der Schaar M. Krljanac G. (2021). Smoking and sex differences in first manifestation of cardiovascular disease. Atherosclerosis 330, 43–51. 10.1016/J.ATHEROSCLEROSIS.2021.06.909 34233252

[B111] Verschoor C. P. Lin D. T. S. Kobor M. S. Mian O. Ma J. Pare G. (2021). Epigenetic age is associated with baseline and 3-year change in frailty in the Canadian Longitudinal Study on Aging. Clin. Epigenetics 13, 163. 10.1186/s13148-021-01150-1 34425884 PMC8381580

[B112] Villar-Fincheira P. Sanhueza-Olivares F. Norambuena-Soto I. Cancino-Arenas N. Hernandez-Vargas F. Troncoso R. (2021). Role of interleukin-6 in vascular health and disease. Front. Mol. Biosci. 8, 79. 10.3389/fmolb.2021.641734 PMC8004548 33786327

