# Supplementary material for: Sex differences in epigenetic age in Mediterranean high longevity regions
Source: Front Aging. 2022 Nov 23;3:1007098. doi: 10.3389/fragi.2022.1007098 (PMC9726738; doi:10.3389/fragi.2022.1007098)
Supplement: Supplementary file 1 [file Table1.docx]

**Supplementary Table 1.** Outline of DNA-methylation biomarkers used in the assessment of sex differences in men and women of the Mediterranean Blue Zones

| **Clock** | **Source** | **Number of CpGs** | **Output** | **Units** | **Mean Age (Age Range) years** | **Training Cohort/Data** | **Additional Variables Included in Predictor** |
| --- | --- | --- | --- | --- | --- | --- | --- |
| **Horvath Pan-Tissue Clock** | Horvath, 2013 | 353 | Chronological age | Years | 43 (0 – 93) | GSE41037, GSE41169,  GSE40279, GSE36064,  GSE32149, GSE27317,  GSE38873, GSE15745,  Brain Cloud, GSE41826,  GSE32393, GSE25892,  GSE20242, GSE42700,  GSE43269, TCGA,  GSE32146, GSE22595,  E-MTAB-202, GSE30601,  GSE42510, GSE37988,  GSE17448, GSE36642,  GSE26126, GSE34035,  GSE28746, GSE20067,  GSE20236, GSE19711,  GSE42861, GSE27097,  GSE30870, GSE37008,  GSE36812, GSE34257,  GSE34639, GSE42865,  GSE38608, GSE38291,  GSE26033, GSE36166,  GSE44667, GSE30758,  GSE40700, GSE26033,  GSE41782, GSE26974,  GSE47627, GSE30653,  GSE31848, GSE37066,  GSE30090, GSE30456,  GSE35069 |  |
| **Hannum** | Hannum *et al.*, 2013 | 71 | Chronological age | Years | (19 – 101) | Novel experimental data |  |
| **GrimAge** | Lu, Quach, *et al.*, 2019 | 1030 | Phenotypic age | Years | 66 | Framingham Heart Study | DNAm-predictors of adrenomedullin, C-reactive protein, plasminogen activation inhibitor 1 (PAI-1), growth differentiation factor 15 (GDF15), leptin, tissue inhibitor metalloproteinase 1 (TIMP-1), Cystatin C, beta-2 microglobulin, DNAm estimator of smoking pack-years |
| **PhenoAge** | Levine *et al.*, 2018 | 513 | Phenotypic age | Years | (21 – 100) | NHANES III for phenotypic age, InCHIANTI for clock | Albumin, creatinine, serum glucose, C-reactive protein (log), lymphocyte percent, mean red cell volume, red cell distribution width, alkaline phosphatase, white blood cell count |
| **Skin and Blood** | Horvath *et al.*, 2018 | 391 | Chronological age | Years | (0 – 94) | GSE79056, SkinE-MTAB-4385, GSE52026, GSE77136, GSE104471, GSE50759, GSE80261, and novel experimental data | Human primary dermal fibroblast cell lines were  obtained from The Progeria Research Foundation (PRF) Cell and Tissue Bank; Primary human skin keratinocytes, fibroblasts and microvascular endothelial cells were isolated from  neonatal foreskin and adult facial/neck skin |
| **DunedinPACE** | Belsky *et al.*, 2022 | 173 | Rate of decline per year | Rate of physiological decline | (26 – 45) | Dunedin birth cohort | albumin, alkaline phosphatase (log), blood urea nitrogen, creatinine (log), C-reactive protein (log), HbA1C, systolic blood pressure, and forced expiratory volume in 1 second (FEV1) |
| **DNAmTL** | Lu, Seeboth, *et al.*, 2019 | 140 | Leukocyte telomere length | Kilobases | (22 – 93) | Framingham Heart Study offspring cohort, Women’s Health Initiative and Jackson Heart Study cohort |  |
| **IL-6 Score** | Stevenson *et al.*, 2021 | 35 |  | (numeric) | 70 | Lothian Birth Cohort 1936 |  |
| **EpiSmokEr** | Bollepalli *et al.*, 2019 | 187 |  | (numeric) | 52 (25 – 74) | Finnish DILGOM cohort |  |

**References**

Belsky, D. W. *et al.* (2022) ‘DunedinPACE, A DNA methylation biomarker of the Pace of Aging’, *eLife*. eLife Sciences Publications Ltd, 11. doi: 10.7554/ELIFE.73420.

Bollepalli, S. *et al.* (2019) ‘EpiSmokEr: a robust classifier to determine smoking status from DNA methylation data’, *Epigenomics*. Future Medicine Ltd London, UK, 11(13), pp. 1469–1486. doi: 10.2217/epi-2019-0206.

Hannum, G. *et al.* (2013) ‘Genome-wide Methylation Profiles Reveal Quantitative Views of Human Aging Rates’, *Molecular Cell*, 49(2), pp. 359–367. doi: 10.1016/j.molcel.2012.10.016.

Horvath, S. (2013) ‘DNA methylation age of human tissues and cell types’, *Genome Biology*. New York, New York, USA: ACM Press, 14(10), p. R115. doi: 10.1186/gb-2013-14-10-r115.

Horvath, S. *et al.* (2018) ‘Epigenetic clock for skin and blood cells applied to Hutchinson Gilford Progeria Syndrome and ex vivo studies’, *Aging*. Aging (Albany NY), 10(7), pp. 1758–1775. doi: 10.18632/AGING.101508.

Levine, M. E. *et al.* (2018) ‘An epigenetic biomarker of aging for lifespan and healthspan’, *Aging*, 10(4), pp. 573–591. doi: 10.18632/aging.101414.

Lu, A. T., Seeboth, A., *et al.* (2019) ‘DNA methylation-based estimator of telomere length’, *Aging*. Aging (Albany NY), 11(16), pp. 5895–5923. doi: 10.18632/AGING.102173.

Lu, A. T., Quach, A., *et al.* (2019) ‘DNA methylation GrimAge strongly predicts lifespan and healthspan’, *Aging*, 11(2), pp. 303–327. doi: 10.18632/aging.101684.

Stevenson, A. J. *et al.* (2021) ‘Creating and Validating a DNA Methylation-Based Proxy for Interleukin-6’, *The Journals of Gerontology: Series A*. Edited by A. B. Newman. Oxford Academic, 76(12), pp. 2284–2292. doi: 10.1093/gerona/glab046.
