# Supplementary figures and images for: Sex differences in epigenetic age in Mediterranean high longevity regions
Source: Front Aging. 2022 Nov 23;3:1007098. doi: 10.3389/fragi.2022.1007098 (PMC9726738; doi:10.3389/fragi.2022.1007098)

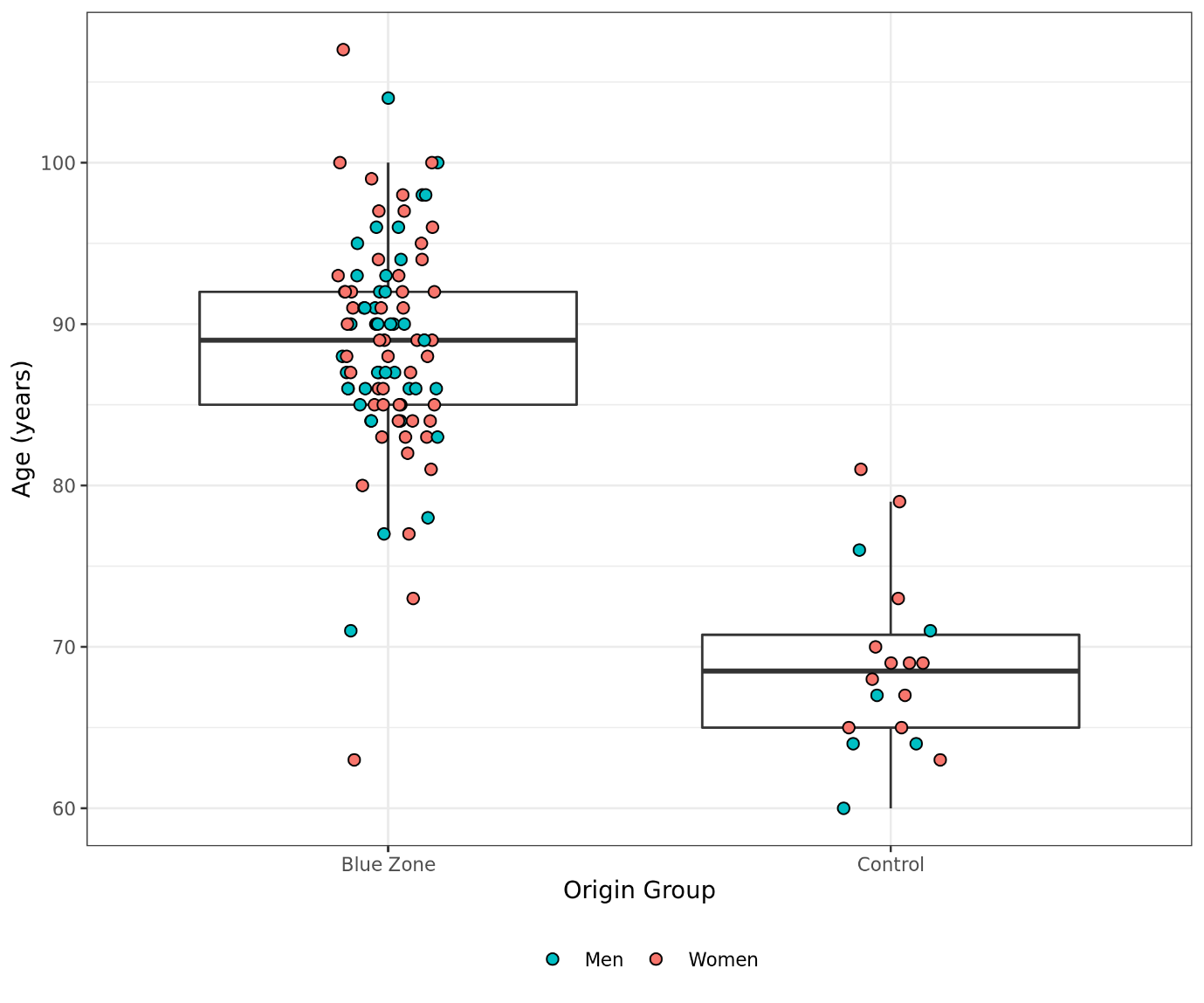

Supplement: Supplementary file 2 [file Image1.tif]
