# Supplementary material for: Sex differences in epigenetic age in Mediterranean high longevity regions
Source: Front Aging. 2022 Nov 23;3:1007098. doi: 10.3389/fragi.2022.1007098 (PMC9726738; doi:10.3389/fragi.2022.1007098)
Supplement: Supplementary file 3 [file DataSheet1.docx]

Supplementary Material

# Supplementary Data

Supplementary Material should be uploaded separately on submission. Please include any supplementary data, figures and/or tables. All supplementary files are deposited to FigShare for permanent storage and receive a DOI.

Supplementary material is not typeset so please ensure that all information is clearly presented, the appropriate caption is included in the file and not in the manuscript, and that the style conforms to the rest of the article. To avoid discrepancies between the published article and the supplementary material, please do not add the title, author list, affiliations or correspondence in the supplementary files.

# Supplementary Figures and Tables

For more information on Supplementary Material and for details on the different file types accepted, please see [here](http://home.frontiersin.org/about/author-guidelines#SupplementaryMaterial). Figures, tables, and images will be published under a Creative Commons CC-BY licence and permission must be obtained for use of copyrighted material from other sources (including re-published/adapted/modified/partial figures and images from the internet). It is the responsibility of the authors to acquire the licenses, to follow any citation instructions requested by third-party rights holders, and cover any supplementary charges.

**Normalization Methods and Epigenetic Clock Predictions**

Normalization and pre-processing of DNA methylation (DNAm) array data is required to match the detected methylation distribution of the two different types of probes present on the array, as well as remove unwanted background or technical variation (Fortin, Triche and Hansen, 2017). There are many different types of normalization that have been developed and adapted to Illumina DNAm arrays. There is currently no consensus regarding what types of data normalization are most appropriate for epigenetic age prediction. Quantile normalization has previously been shown to increase the predicted epigenetic age more than Illumina Genome Studio normalization, and noob background correction, but predicted epigenetic age remained highly correlated across all methods, and epigenetic age acceleration (EAA) remains relatively unaffected by normalization choice (McEwen *et al.*, 2018). In some cases, studies have chosen different normalization methods per clock (Verschoor *et al.*, 2021). In this study, noob background correction (Triche *et al.*, 2013), BMIQ normalization (with ComBat batch correction) (Johnson, Li and Rabinovic, 2007; Teschendorff *et al.*, 2013), SWAN (with ComBat batch correction) (Maksimovic, Gordon and Oshlack, 2012), and functional normalization (funnorm) (Fortin *et al.*, 2014) with ComBat batch correction were compared to each other using the functions in the *minfi* R package (Aryee *et al.*, 2014; Fortin, Triche and Hansen, 2016). The chosen method for analysis was funnorm and ComBat correction as the normalization method. This choice was made based on the prediction similarity between the Horvath and Hannum clocks, both of which were trained to predict chronological age without other health information (Hannum *et al.*, 2013; Horvath, 2013; Levine *et al.*, 2018; Lu *et al.*, 2019), and had smaller sex discrepancies in Pearson correlation (*r*) (Supplementary Figures 2 and 3).

The variation between most clocks was reduced by funnorm and ComBat correction. The exception in this case was PhenoAge prediction, which became more accurate at predicting older chronological age with funnorm and ComBat preprocessing steps. EAA remained greater in men than women across preprocessing methods, and all preprocessing methods resulted in stronger correlations of predicted epigenetic age and chronological age in women rather than men.


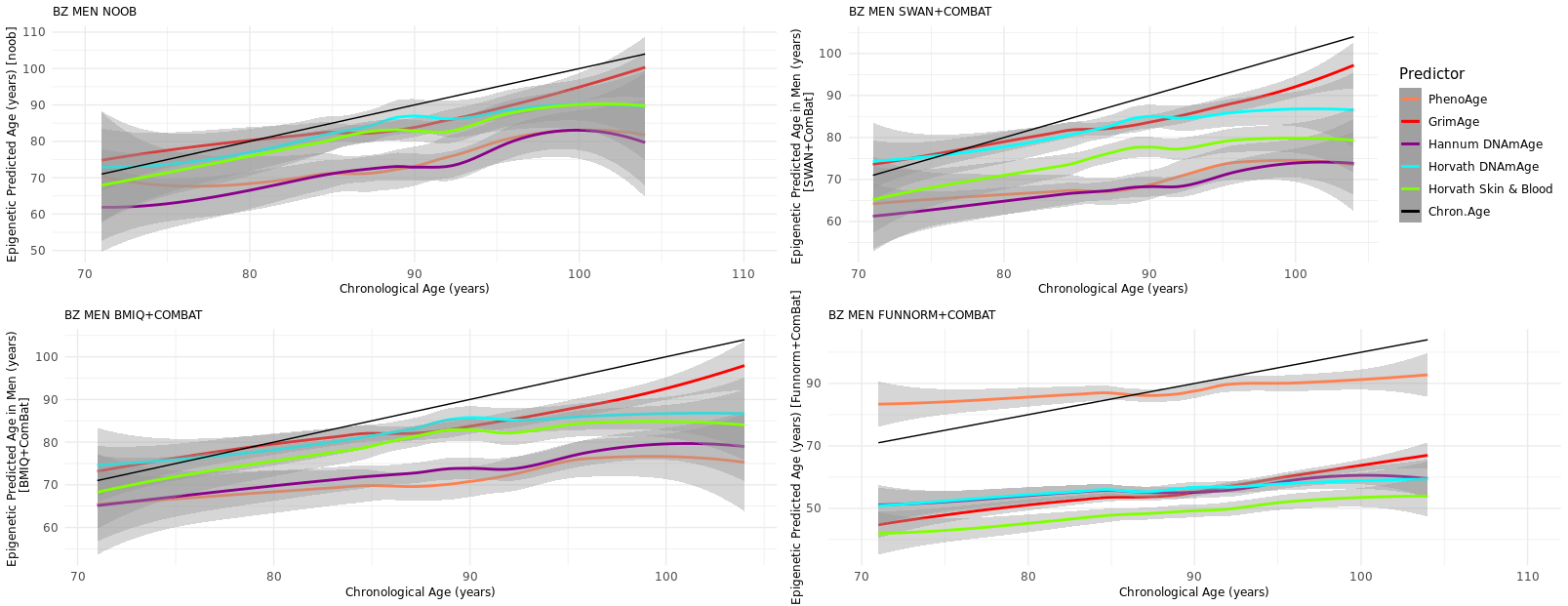


**A**

**B**

**D**

**C**

**Supplementary Figure 2.** A Loess-smoothed curve of epigenetic predicted age in men from the Mediterranean Blue Zones with 95% confidence interval in grey against chronological age in years according to the PhenoAge, GrimAge, Hannum, Horvath, and Skin and Blood epigenetic age predictors using either A) noob background correction, B) SWAN and ComBat correction, C) BMIQ and ComBat correction, or D) funnorm and ComBat correction.


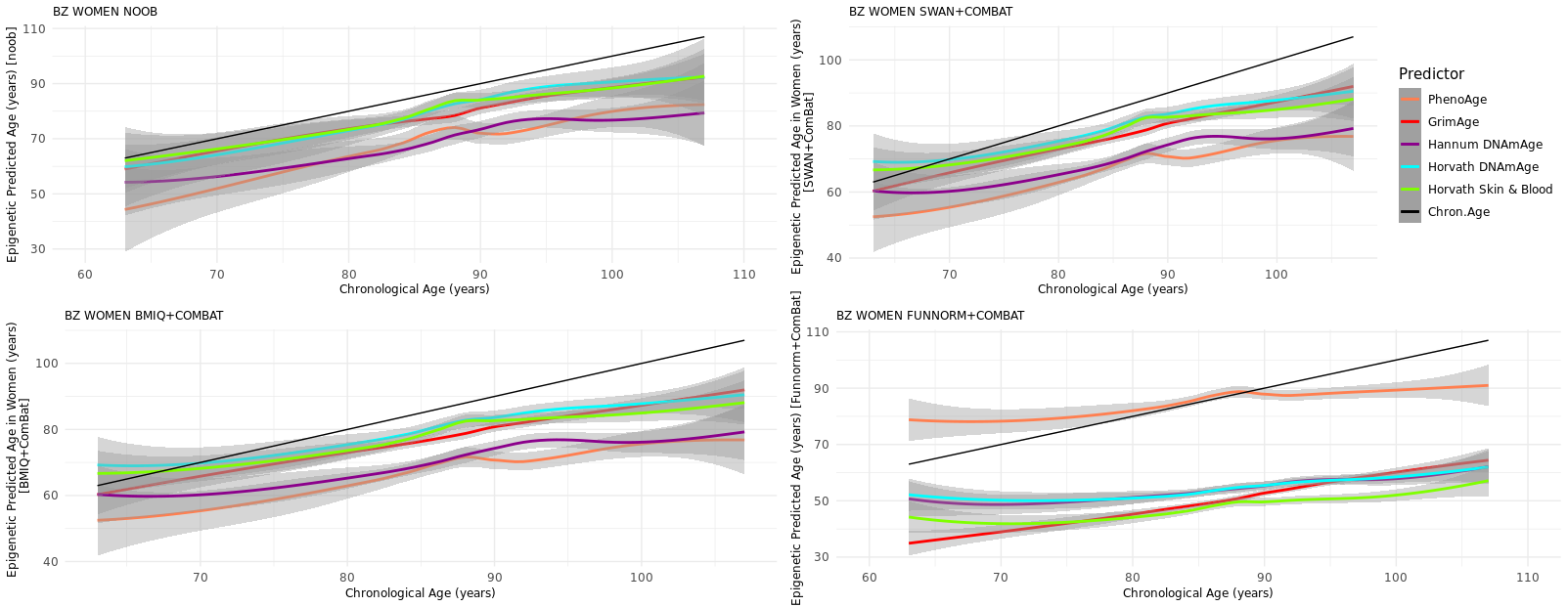


**D**

**C**

**A**

**B**

**Supplementary Figure 3.** A Loess-smoothed curve of epigenetic predicted age in women from the Mediterranean Blue Zones with 95% confidence interval in grey against chronological age in years according to the PhenoAge, GrimAge, Hannum, Horvath, and Skin and Blood epigenetic age predictors using either A) noob background correction, B) SWAN and ComBat correction, C) BMIQ and ComBat correction, or D) funnorm and ComBat correction.

**A**

**B**

**References**

Aryee, M. J. *et al.* (2014) ‘Minfi: a flexible and comprehensive Bioconductor package for the analysis of Infinium DNA methylation microarrays’, *Bioinformatics*, 30(10), pp. 1363–1369. doi: 10.1093/bioinformatics/btu049.

Fortin, J.-P. *et al.* (2014) ‘Functional normalization of 450k methylation array data improves replication in large cancer studies’, *Genome Biology*, 15(11), p. 503. doi: 10.1186/s13059-014-0503-2.

Fortin, J.-P., Triche, T. J. and Hansen, K. D. (2016) ‘Preprocessing, normalization and integration of the Illumina HumanMethylationEPIC array with minfi’, *Bioinformatics*, p. btw691. doi: 10.1093/bioinformatics/btw691.

Fortin, J. P., Triche, T. J. and Hansen, K. D. (2017) ‘Preprocessing, normalization and integration of the Illumina HumanMethylationEPIC array with minfi’, *Bioinformatics*. Oxford Academic, 33(4), pp. 558–560. doi: 10.1093/BIOINFORMATICS/BTW691.

Hannum, G. *et al.* (2013) ‘Genome-wide Methylation Profiles Reveal Quantitative Views of Human Aging Rates’, *Molecular Cell*, 49(2), pp. 359–367. doi: 10.1016/j.molcel.2012.10.016.

Horvath, S. (2013) ‘DNA methylation age of human tissues and cell types’, *Genome Biology*. New York, New York, USA: ACM Press, 14(10), p. R115. doi: 10.1186/gb-2013-14-10-r115.

Johnson, W. E., Li, C. and Rabinovic, A. (2007) ‘Adjusting batch effects in microarray expression data using empirical Bayes methods’, *Biostatistics (Oxford, England)*. Biostatistics, 8(1), pp. 118–127. doi: 10.1093/BIOSTATISTICS/KXJ037.

Levine, M. E. *et al.* (2018) ‘An epigenetic biomarker of aging for lifespan and healthspan’, *Aging*, 10(4), pp. 573–591. doi: 10.18632/aging.101414.

Lu, A. T. *et al.* (2019) ‘DNA methylation GrimAge strongly predicts lifespan and healthspan’, *Aging*, 11(2), pp. 303–327. doi: 10.18632/aging.101684.

Maksimovic, J., Gordon, L. and Oshlack, A. (2012) ‘SWAN: Subset-quantile within array normalization for illumina infinium HumanMethylation450 BeadChips.’, *Genome biology*. BioMed Central, 13(6), pp. 1–12. doi: 10.1186/GB-2012-13-6-R44/FIGURES/7.

McEwen, L. M. *et al.* (2018) ‘Systematic evaluation of DNA methylation age estimation with common preprocessing methods and the Infinium MethylationEPIC BeadChip array’, *Clinical Epigenetics*, 10(1), p. 123. doi: 10.1186/s13148-018-0556-2.

Teschendorff, A. E. *et al.* (2013) ‘A beta-mixture quantile normalization method for correcting probe design bias in Illumina Infinium 450 k DNA methylation data’, 29(2), pp. 189–196. doi: 10.1093/bioinformatics/bts680.

Triche, T. J. *et al.* (2013) ‘Low-level processing of Illumina Infinium DNA Methylation BeadArrays’, *Nucleic Acids Research*. Oxford University Press, 41(7), p. e90. doi: 10.1093/NAR/GKT090.

Verschoor, C. P. *et al.* (2021) ‘Epigenetic age is associated with baseline and 3-year change in frailty in the Canadian Longitudinal Study on Aging’, *Clinical Epigenetics*. BioMed Central Ltd, 13(1), pp. 1–10. doi: 10.1186/S13148-021-01150-1/TABLES/2.
